# Low-penetrance TP53 variants are mainly hypomorphic: an underestimated issue with high clinical significance

**DOI:** 10.1038/s41525-026-00568-x

**Published:** 2026-04-18

**Authors:** Lea Rodriguez, Bernard Leroy, Franck Toledo, Julianne Susanne Funk, Thorsten Stiewe, Panagiotis Baliakas, François Delhommeau, Thierry Soussi

**Affiliations:** 1 Sorbonne Université, INSERM, Centre de Recherche Saint-Antoine, CRSA, UMRS_938 Hematopoietic and Leukemic Development, AP-HP, SIRIC CURAMUS, Paris, France; 2 Carnot OPALE Institute, Paris, France; 3 Institute of Molecular Oncology, Universities of Gießen and Marburg Lung Center (UGMLC), Member of the German Center for Lung Research (DZL), Philipps-University, Marburg, Germany; 4 Institute of Lung Health, Justus Liebig University, Gießen, Germany; 5 Department of Immunology, Genetics and Pathology, Uppsala University, Uppsala, Sweden; 6 Clinical Genomics Uppsala, Science for Life Laboratory, Uppsala University, Uppsala, Sweden

**Keywords:** Cancer, Computational biology and bioinformatics, Genetics, Oncology

## Abstract

Missense mutations that inactivate p53 are common in cancer. LiFraumeni syndrome, which is linked to early-onset cancer, is caused by germline mutations in *TP53*. Full-penetrant, inactive variants have garnered great attention, whereas low-penetrant variants are less well understood despite their clinical importance. This study systematically leveraged the 2025 UMD_TP53 database to identify missense variants that exhibit a statistically skewed germline-versus-somatic ratio (GVSr). Unlike classic hotspots that are equally prevalent in somatic and germline settings, these variants were disproportionately found in the germline, suggesting they act as low-penetrance variants with insufficient potency to drive tumorigenesis as single somatic events. To define the biological basis of LPVs, we integrated functional data from multiplexed assays of variant effects, tumor cell transcriptome analyses and computational predictive tools. This characterization revealed that these high-GVSr p53 variants consistently retain intermediate transcriptional activity and growth-suppressive function, classifying them distinctively as hypomorphic alleles rather than loss-of-function mutants. Our findings highlight the complexity of *TP53* variant effects and underscore the importance of refined functional classification. Recognizing and accurately characterizing hypomorphic variants associated with low-penetrance cancer risk are essential for precision oncology, as they will improve genetic counseling, risk stratification, and tailored surveillance strategies for individuals with *TP53* mutations.

## Introduction

*TP53* is classified as a tumor suppressor gene, but more than 80% of p53 alterations are missense variants scattered along the entire protein with a predominance in the DNA-binding domain and tetramerization domains^1,2^. *TP53* germline mutation predisposes individuals to hereditary cancer syndromes, such as Li-Fraumeni syndrome (LFS). However, recent studies have shown that, depending on the p53 variants, these germline mutations can be identified outside the context of classic LFS diagnosis, leading to a broader spectrum of cancer risks and phenotypes^3^. Somatic or germline p53 variants resulting from these mutations are associated with a loss of tumor suppressive activity, but it has been established that this situation is highly heterogeneous. True amorphic variants, generally associated with nonsense or frameshift mutations, are typically easy to classify. However, it is widely accepted that missense variants are highly heterogeneous, with full or partial loss of function being not only variable across the different variants but also dependent on the cellular context^2,4^. Furthermore, two other non-exclusive characteristics of p53 variants add another layer of complexity. First, because the p53 protein is active as a tetramer, antimorphic variants (also known as dominant negative (DN) variants) lead to the disruption of wild-type activity through hetero-oligomerization with the wild-type variant^5^. Although the scientific community widely accepts the specific effect of these p53 variants, any clinical applications concerning it are still pending. The second layer of complexity is the identification of neomorphic variants where p53 exerts novel oncogenic activities and promotes cell transformation. These “gain-of-function” (GOF) variants remain highly debated and far from clinical application^6–8^.

A less-explored field with potent and immediate clinical applications is hypomorphic variants that result in either a reduced global loss of protein activity or a function-specific defect. p53 p.R175P was the first variant shown to have heterogeneous loss of activity, as it retains the ability to induce cell cycle arrest but loses the capacity to trigger apoptosis, and is less tumorigenic in animal models^9–11^.

In 2005, we used a clustering method to assign *TP53* missense variants frequently found in human tumors based on their functional activities in experimental assays assessing biological p53 functions performed in yeast. That work led to the identification of multiple hypomorphic variants, including several at codons 181, 290, or 235^12^. Later studies showed that some of these variants, such as p.R290H and p.N235S, were indeed benign SNPs inadvertently defined as cancer-associated variants^13^. Contrastingly though, some of these hypomorphic variants, such as p.R181H and p.R181C, were shown to be associated with a partial loss of their tumor suppressive activity but also increased sensitivity for apoptotic chemotherapeutic agents as compared to classic hotspot variants^14–16^.

In parallel with these functional studies, genetic analyses of populations at risk of cancer have identified p53 variants associated with low familial penetrance and particular cancer profiles^17–22^. Functional analysis of these variants has revealed that they are all hypomorphic with specific characteristics that differentiate them from classic hotspot variants. Among them, families with germline variants at codon 181 (p.R181H and p.R181C) are predominantly associated with breast cancer. Similarly, germline variants in the oligomerization domain (p.R337H and p.G334R), which are associated with an increased cancer risk, were also shown to be partially defective. Taken together, these observations indicate a link between the heterogeneity of p53 loss of activity and the penetrance of these variants in familial cancer.

In the present study, using the UMD_TP53 database, we identified a subset of p53 variants detected in both somatic and germline settings that are statistically overrepresented as germline alterations relative to classic hotspot mutations. Further analyses demonstrated that these variants constitute a distinct class of hypomorphic alleles associated with partial loss of function and low cancer penetrance in humans.

## Results

### Germline-versus-somatic ratio reveals atypical-penetrance variants

In a previous work, we discovered that benign, infrequent, single-nucleotide polymorphisms (SNPs), including ethnic variants specific to Asian or African populations, were included in all *TP53* locus-specific databases (LSBDs)^13,23^. That analysis assumed that rare SNPs could be mistakenly identified as somatic variants in human tumors. Indeed, in many studies, normal tissue is generally not available and assessments of variant origin are often compared to the reference human genome and the Single Nucleotide Polymorphism Database (dbSNP), this latter providing a limited number of *TP53* population variants. *TP53* mutation can be identified both as somatic variants in all cancer types and as a germline variant associated with Li-Fraumeni, a cancer predisposition syndrome associated with high risks for a broad spectrum of cancers. For that reason, *TP53* LSDBs include both types of variants^24,25^. Furthermore, the landscapes of somatic and germline TP53 mutations are similar, sharing the same hotspot variants^1^. The germline-versus-somatic ratio (GVSr) developed by Doffe et al. defines the frequency at which each *TP53* variant can be found as germline. For most p53 variants, including the most common ones associated with LFS, this ratio is very low (<5). The variants found at high GVSr by Doffe et al. were indeed genuine SNPs, and they have been tagged accordingly in the database^13,23^.

**Fig. 1:**
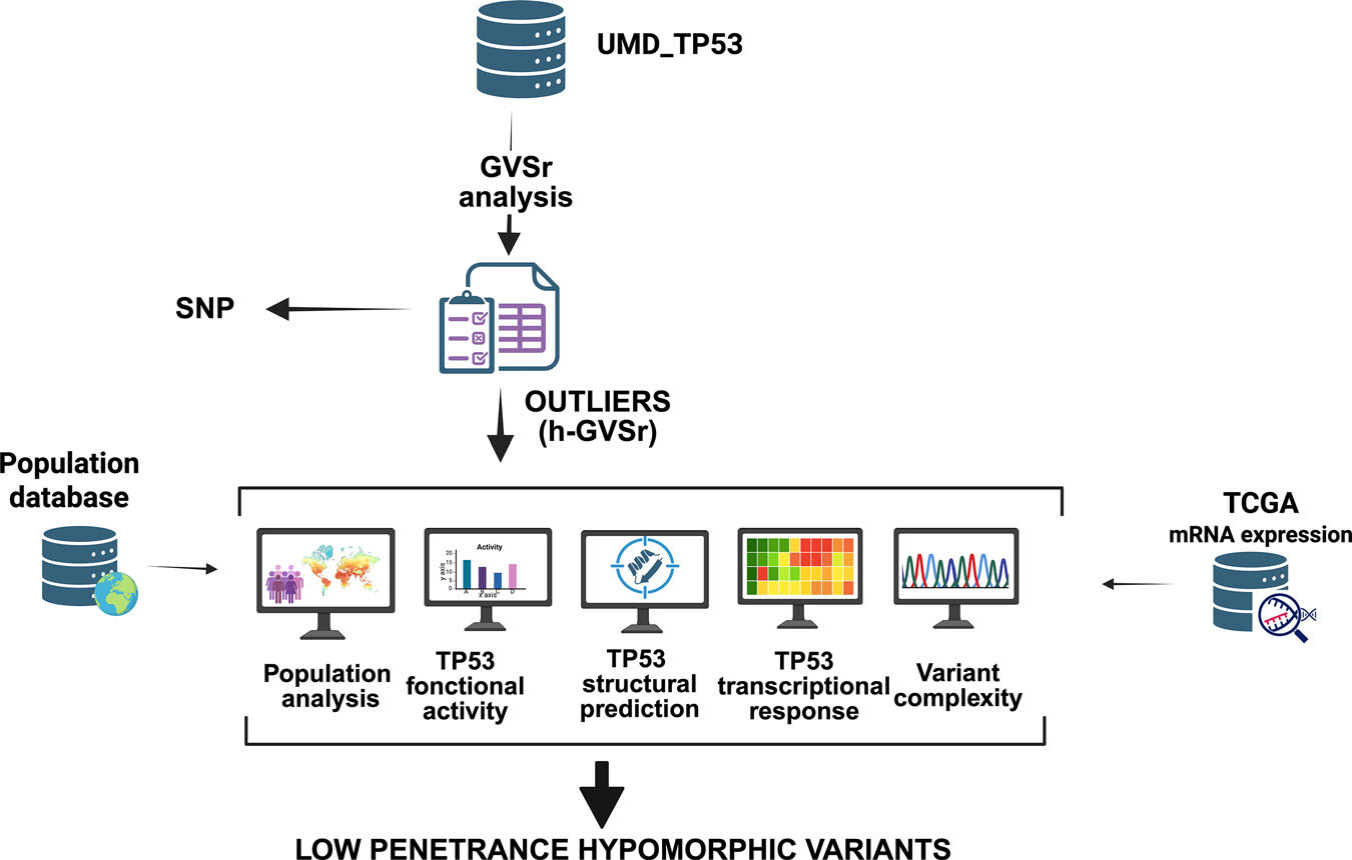
Flowchart of the strategy to identify potential novel *TP53* hypomorphic variants. Three different datasets were employed for missense variants: the UMD_TP53 database, the UMD_TP53_SNP database, and the GENIE data registry (see “Methods” for further information).

**Fig. 2:**
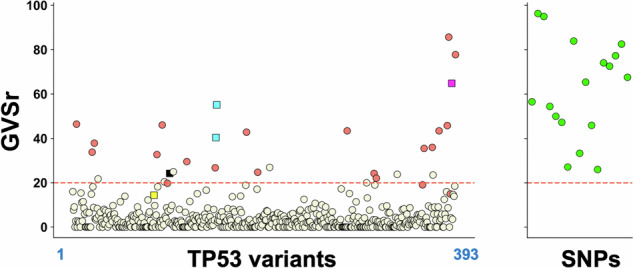
Germline-versus-somatic ratio (GVSr) of *TP53* variants in UMD_TP53. Left: Analysis on missense *TP53* variants with at least 20 occurrences in the database. Variants are arranged from left to right according to their positions in the p53 protein (residues 1 to 393). Right: the validated missense SNPs identified by Doffe et al. are shown in green^13^. h-GVSr are shown in orange. Hypomorphic variants at codons 152, 158, 181, and 337 are shown in yellow, black, blue, and purple, respectively. Most statistically significant h-GVSr variants are above 20 (red line).

We performed a novel GVSr analysis on the updated release of UMD_TP53 (Figs. 1 and 2) (see “Methods” for a description of UMD_TP53). GVSr was lower than 3 (median 2.73, interquartile range 3.45) for the great majority of p53 variants, including p53 hotspot variants (Fig. 2 and Supplementary Fig. S1). Seventeen of the previously identified SNPs, all with high GVSr, were still included in numerous publications, despite the fact that seven of them have been validated by the FDA as true benign polymorphisms (median 47.05, IQR 44.10) (Fig. 2 and Table 1)^23,26^. The GVSr analysis also identified a skewed, high-GVSr distribution for a new set of p53 variants (Fig. 2 and Supplementary Fig. S1). In that set, we remarked five variants (p.R181C/H, p.R337H, p.P152L, or p.R158H) previously identified as lowpenetrance p53 variants associated with a reduced risk of cancer^17,19,20^. One explanation for this reported lowpenetrance was that these five missense variants exhibited only partial loss of function and were classified as hypomorphic^12,14,27^. Allele frequency analysis in four independent population databases confirmed that these variants were either very rare or absent in the human population (Supplementary Fig. S2).

**Table 1:** h-GVSr p53 variants identified in this study

cDNA_variant NM_000546	Protein_variant NP_000537	Classification^a^	rs_ID	Clinvar_class	GVSr	Fortunato hypomorphic^b^	Montellier Class^c^	Cluster Kato^d^	p_value_raw (20)^e^
c.31 G > C	p.Glu11Gln	SNP	rs201382018	CCP	96.31		D	1	0
c.91 G > A	p.Val31Ile	SNP	rs201753350	CCP	94.96	–	D	1	0
c.847 C > T	p.Arg283Cys	SNP	rs149633775	LB; FDA approved	45.92	–	D	2	1.25495E-44
c.704 A > G	p.Asn235Ser	SNP	rs144340710	B; FDA approved	65.38	–	D	1	3.06114E-82
c.566 C > T	p.Ala189Val	SNP	rs121912665	CCP	83.91	–	C	1	7.666E-128
c.869 G > A	p.Arg290His	SNP	rs55819519	B; FDA approved	26.00	–	D	1	1.1802E-07
c.214 C > G	p.Pro72Ala	SNP	rs587782769	LB; FDA approved	54.43	–	D	1	5.83146E-26
c.466 C > T	p.Arg156Cys	SNP	rs563378859	CCP	27.12	–	C	2	1.72252E-05
c.329 G > A	p.Arg110His	SNP	rs11540654	LB; FDA approved	47.27	–	C	1	2.45929E-14
c.1079 G > C	p.Gly360Ala	SNP	rs35993958	B; FDA approved	82.54	–	C	1	1.31343E-45
c.665 C > T	p.Pro222Leu	SNP	rs146340390	CCP	33.33	–	D	2	4.11337E-06
c.1015 G > A	p.Glu339Lys	SNP	rs17882252	CCP	72.55	–	D	1	3.79524E-29
c.1073 A > T	p.Glu358Val	SNP	rs773553186	CCP	77.27	–	C	1	1.80533E-28
c.1096 T > G	p.Ser366Ala	SNP	rs17881470	B; FDA approved	67.57	–	D	1	6.33169E-19
c.935 C > G	p.Thr312Ser	SNP	rs145151284	B; FDA approved	74.07	–	D	1	1.18564E-16
c.28 G > A	p.Val10Ile	SNP	rs535274413	B; FDA approved	56.52	–	D	1	4.54455E-09
c.217 G > A	p.Val73Met	SNP	rs587782423	B; FDA approved	50.00	–	D	1	3.80936E-07
c.473 G > A	p.Arg158His	h-GVSr	rs587782144	P/LP	24.22	–	B	3	6.39272E-28
c.455 C > T	p.Pro152Leu	h-GVSr	rs5877827050	P; FDA approved	14.42	–	B	3	1.60449E-05
c.1009 C > T	p.Arg337Cys	h-GVSr	rs5877825290	P; FDA approved	14.99	–	A	3	2.61055E-05
c.638 G > A	p.Arg213Gln	h-GVSr	rs5877787200	P/LP	24.74	–	A	3	2.81358E-17
c.542 G > A	p.Arg181His	h-GVSr	rs397514495	P/LP	55.16	Yes	C	2	7.18194E-91
c.799 C > T	p.Arg267Trp	h-GVSr	rs55832599	P/LP	24.22	Yes	B	3	6.07098E-13
c.375 G > A	p.Thr125=	h-GVSr	rs558636390	P/LP	37.89	–	N.A.	N.A.	3.26383E-25
c.541 C > T	p.Arg181Cys	h-GVSr	rs587782596	CCP	40.41	Yes	C	2	4.28301E-34
c.374 C > T	p.Thr125Met	h-GVSr	rs786201057	CCP	33.82	–	A	3	3.92609E-17
c.1010 G > A	p.Arg337His	h-GVSr	rs121912664	P/LP	64.90	Yes	C	1	1.65843E-67
c.845 G > A	p.Arg282Gln	h-GVSr	rs730882008	CCP	19.09	Yes	C	2	0.000260429
c.472 C > T	p.Arg158Cys	h-GVSr	rs5877800680	CCP	19.78	–	C	2	0.000444988
c.467 G > A	p.Arg156His	h-GVSr	rs371524413	VUS; FDA approved	46.09	Yes	C	2	5.17416E-27
c.848 G > A	p.Arg283His	h-GVSr	rs371409680	VUS	35.56	Yes	A	3	4.67475E-13
c.800 G > A	p.Arg267Gln	h-GVSr	rs587780075	VUS; FDA approved	22.09	–	C	2	6.74543E-05
c.538 G > A	p.Glu180Lys	h-GVSr	rs879253911	LP; FDA approved	26.76	–	B	2	3.59035E-06
c.460 G > A	p.Gly154Ser	h-GVSr	rs137852789	VUS	32.73	–	B	2	2.45355E-07
c.509 C > T	p.Thr170Met	h-GVSr	rs779000871	LB; FDA approved	29.55	–	C	2	3.89768E-05
c.605 G > A	p.Arg202His	h-GVSr	rs587778719	CCP	42.86	–	C	1	7.69175E-11
c.245 C > T	p.Pro82Leu	h-GVSr	rs534447939	LB; FDA approved	46.43	–	D	1	9.98641E-08
c.1079 G > T	p.Gly360Val	h-GVSr	rs35993958	CCP	77.78	–	C	1	8.1491E-24
c.1000 G > C	p.Gly334Arg	h-GVSr	rs730882028	VUS; FDA approved	85.71	Yes	D	1	1.64107E-22
c.884 C > T	p.Pro295Leu	h-GVSr	rs751713111	CCP	36.00	–	D	1	0.000111212
c.998 G > A	p.Arg333His	h-GVSr	rs573154688	B; FDA approved	45.83	–	D	1	1.1521E-06
c.949 C > A	p.Gln317Lys	h-GVSr	rs764735889	B; FDA approved	43.48	–	D	1	6.42701E-06
c.760 A > G	p.Ile254Val	h-GVSr	rs746601313	LB; FDA approved	43.48	–	D	1	6.42701E-06
c.314 G > A	p.Gly105Asp	h-GVSr (10)	rs587781504	CCP	31.58	–	B	2	–
c.1040 C > A	p.Ala347Asp	h-GVSr (10)	N.A.	P; FDA approved	89.47	–	B	2	–
c.877 G > T	p.Gly293Trp	h-GVSr (10)	rs587780076	LB; FDA approved	35.29	–	D	1	–
c.382 C > A	p.Pro128Thr	h-GVSr (10)	rs1555526327	CCP	82.35	–	D	1	–
c.319 T > C	p.Tyr107His	SNP (10)	rs368771578	B; FDA approved	52.94	–	C	1	–
c.787 A > G	p.Asn263Asp	SNP (10)	rs72661119	CCP	60.00	–	C	2	–
c.1175 C > T	p.Ser392Leu	h-GVSr (10)	rs769664911	N.A.	92.86	–	D	1	–
c.917 G > A	p.Arg306Gln	h-GVSr (10)	rs1048095040	CCP	42.86	–	B	3	–
c.572 C > G	p.Pro191Arg	h-GVSr (10)	rs587778718	LB; FDA approved	71.43	–	D	1	–
c.129 G > C	p.Leu43Phe	h-GVSr (10)	rs754332870	CCP	92.86	–	D	1	–
c.1101-1 G > A	p.?	h-GVSr (10)	rs876658982	P/LP	38.46	Yes	N.A.	N.A.	–
c.1101-2 A > G	p.?	h-GVSr (10)	N.A.	P/LP	61.54	–	N.A.	N.A.	–
c.642 T > G	p.His214Gln	h-GVSr (10)	rs587781386	CCP	53.85	–	D	1	–
c.257 C > T	p.Ala86Val	h-GVSr (10)	N.A.	CCP	38.46	–	D	1	–
c.218 T > C	p.Val73Ala	h-GVSr (10)	N.A.	VUS	38.46	–	D	1	–
c.861 G > T	p.Glu287Asp	h-GVSr (10)	N.A.	VUS	41.67	–	D	1	–
c.698 A > G	p.His233Arg	h-GVSr (10)	rs879254233	CCP	41.67	–	C	1	–
c.248 C > T	p.Ala83Val	SNP (10)	rs201717599	CCP	41.67	–	D	1	–
c.1136 G > A	p.Arg379His	h-GVSr (10)	rs863224682	LB; FDA approved	63.64	–	C	1	–
c.1093 C > T	p.His365Tyr	h-GVSr (10)	rs267605075	LB; FDA approved	63.64	–	C	1	–
c.479 T > C	p.Met160Thr	h-GVSr (10)	N.A.	VUS	54.55	–	D	1	–

^a^Variant classification: *SNP* single nucleotide polymorphism; h-GVSr—high GVSr variants identified in UMD_TP53 using an occurrence threshold of 20 (h-GVSr fully described in the text); h-GVSr (10)—high GVSr variants identified in UMD_TP53 using an occurrence threshold of 10.

^b^Variants defined as “hypomorphic” by Fortuno et al.^62^.

^c^Variant classification defined by Montellier et al.^31^.

^d^Variant classification defined by Soussi et al.^12^.

^e^*p* value from the Fisher test performed on GVSr data using an occurrence threshold of 20. The *p*-value from the Fisher test performed on GVSr data using an occurrence threshold of 10 is shown on supplementary table S1.

**Fig. 3:**
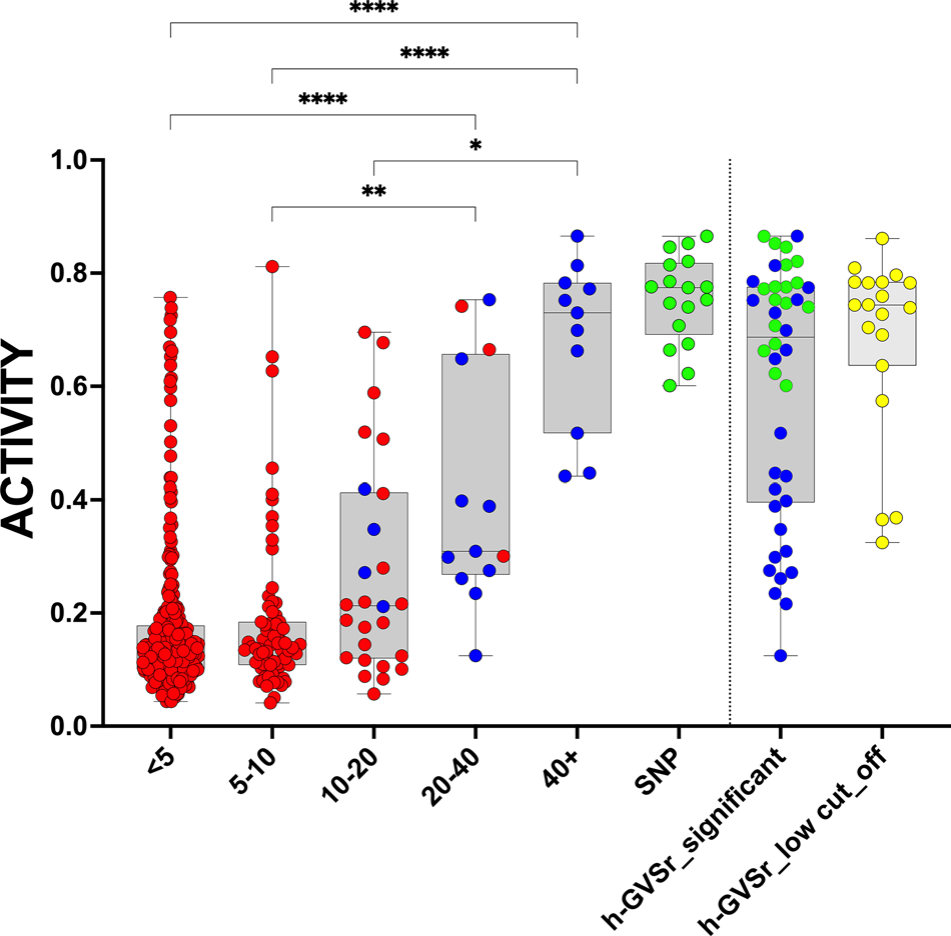
Analysis of the activity of *TP53* variants according to their frequency in UMD_TP53. Left box plots display *TP53* variant loss of function for each *TP53* variant classified into five categories according to their GVSr. Each dot corresponds to a single missense p53 variant. The last category (SNP in green) summarizes the GVSr value of variants previously defined as SNPs^23^. The Y-axis shows variant-normalized *TP53* functionality using all functional datasets (see “Methods”). Variants selected after the statistical analysis are shown in dark blue. The GVSr selection was performed for variants found at a frequency higher than 20 in UMD_TP53. Variants in yellow (right box plot) were identified using a frequency of 10. Synonymous variants at codon 125 known to be associated with splice alteration are not included in this graph. *P*-values were calculated by the Kruskal–Wallis test. **, ***, and ****: *P*  < 0.01, *P* < 0.001 and *P* < 0.0001, respectively.

We therefore investigated the functional activity of the variants identified by the GVSr analysis. We observed a characteristic enrichment of nonfunctional variants in mutants with lowGVSr (Fig. 3). In contrast, previously described SNPs remained highly functional. Moreover, many variants with highGVSr displayed only a partial loss of function, including the five hypomorphic variants described above (Fig. 3). HighGVSr is highly specific for non-hotspot missense variants and not observed for null variants, such as nonsense or frameshift variants (Supplementary Fig. S3A, B). We thus focused our analysis on the functional characterization of high-GVSr variants (h-GVSr hereafter), suspecting that they may be low-penetrant variants associated with partial loss of activity. We performed a statistical analysis on the GVSr results to select 26 variants found more than 20 times in UMD_TP53 (see “Methods”). This statistically significant set included the five hypomorphic variants described previously and all the variants comprised in it had a GVSr above 10 (Fig. 3, Table 1 and Supplementary Table S1).

### h-GVSr p53 variants show increased association with TP53 complexity

**Fig. 4:**
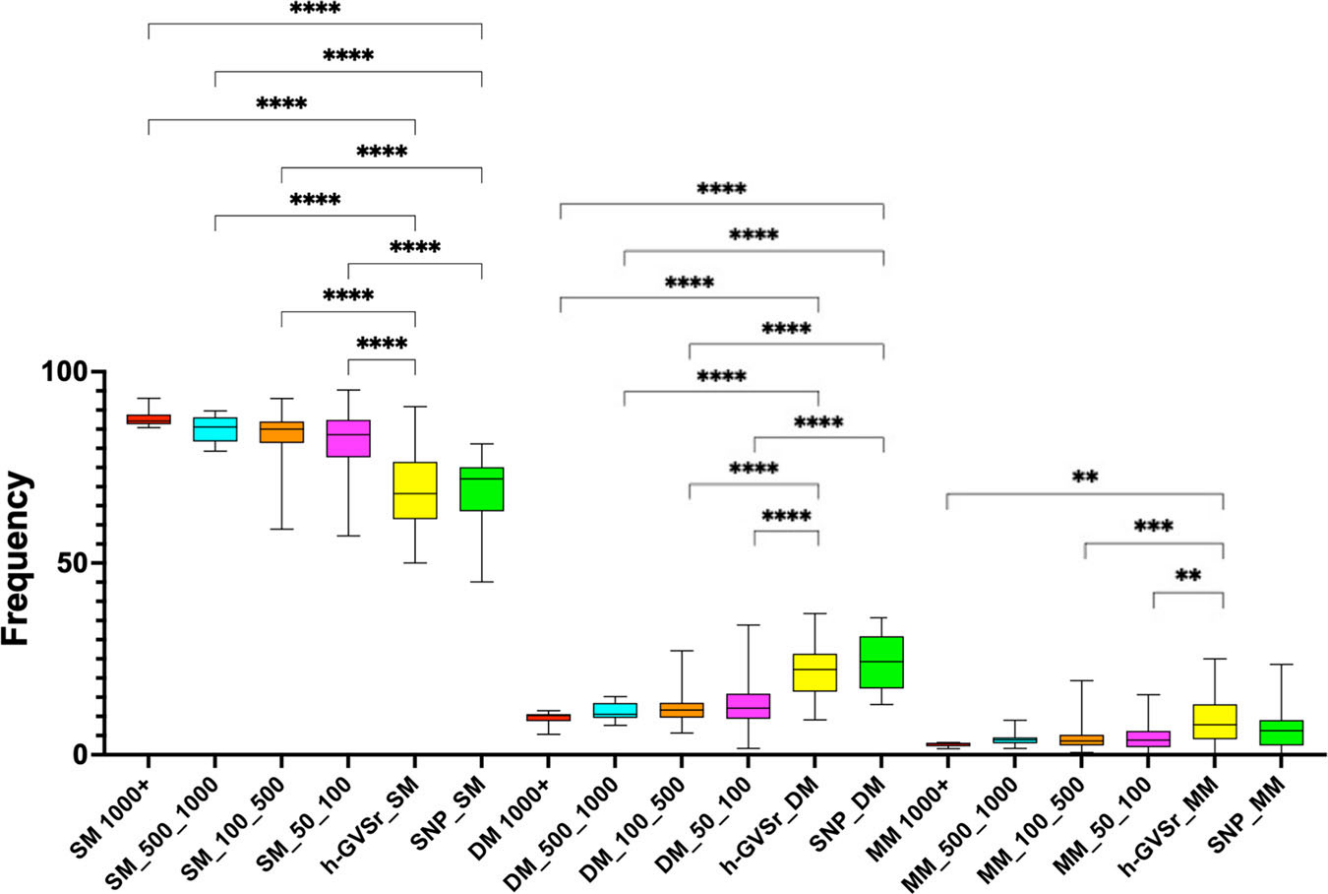
h-GVSr p53 variants are frequently found in tumors expressing multiple p53 variants. Somatic missense *TP53* variants from UMD_TP53 were categorized into four groups based on their frequencies ( >1000, 500_1000, 100_500, and 50_100) and complexity (single, double or multiple mutation, SM, DM, or MM, respectively) in the database. The SNPs are shown separately from the other variants and from h-GVSr variants to avoid potential bias. *P*-values were calculated by the Kruskal–Wallis test. ****P < 0.0001.

The 26 selected h-GVSr variants were evenly distributed as somatic variants in the database, but they also aligned with the most common germline variants. There were no p53 hotspot variants among them (Supplementary Table S2). We reported previously that either SNPs or variants with partial loss of activity were frequently found in tumors with more than one p53 mutation (defined as p53 complexity in this study)^13^. In the case of SNPs, the second mutation was the true driving force selected during tumorigenesis, an aspect that may apply to h-GVSr variants as well. Most p53-mutated tumors in UMD_TP53 express only a single mutation (SM tumors), but tumors with higher *TP53* complexity, expressing either two (DM tumors) or more than two (MM tumors) mutations are also present in the database (Supplementary Fig. S4 and Fig. 4). In UMD_TP53, SM, DM, and MM tumors were found at frequencies of 81%, 12%, and 7%, respectively (Supplementary Fig. S4). Analysis of the 26 h-GVSr variants found higher complexity among them and a greater frequency of association with other p53 variants, in comparison with other cancer-associated variants, independent of variant frequency in the database (Fig. 4). A similar analysis was performed using somatic p53 variants from GENIE, an independent database not included in UMD_TP53. It confirmed the high *TP53* complexity of these h-GVSr variants (Supplementary Fig. S5).

### h-GVSr variants p53 variants display a partial loss of function in multiple large-scale functional analyses

**Fig. 5:**
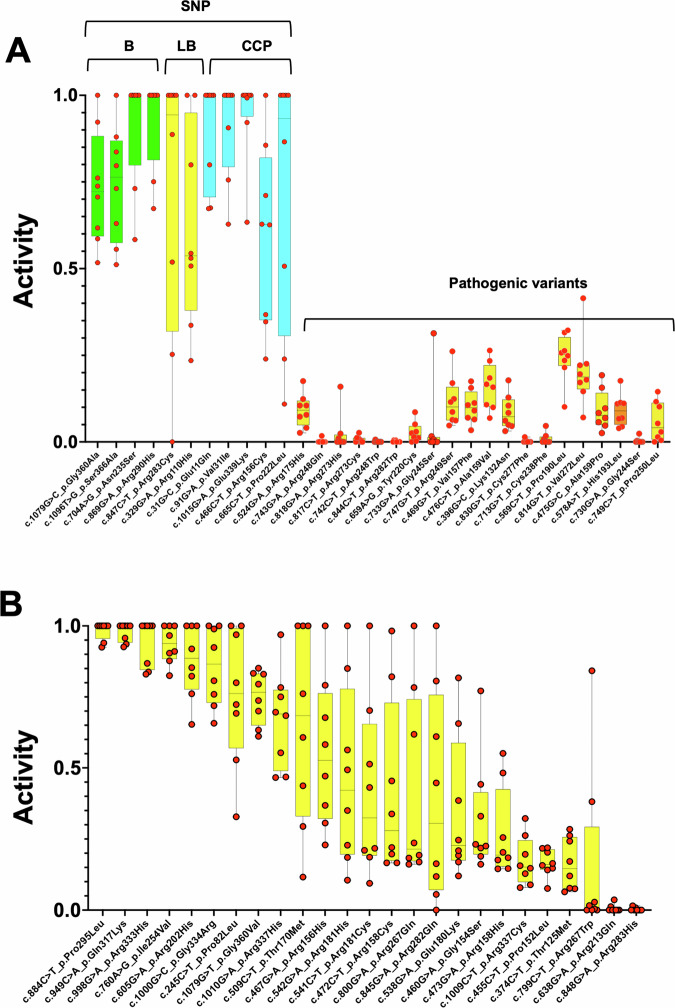
Functional analysis of TP53 variants. **A** pathogenic variants; **B** h-GVSr. Benign (B) and likely benign (LB) are FDA-validated SNPs, whereas conflicting classifications of pathogenicity (CCP) describe variants with conflicting results and no clear validation in dbSNP. Pathogenic variants: the twenty most frequent variants in UMD_TP53. Data for the synonymous variants at codon 125, well known to be also associated with splicing defects, were omitted from all functional analyses. The remaining activity (average functional data on the eight p53 response elements) ranges from 0 (no activity) to 1 (full activity) and was defined from the normalized data of Kato et al.^29^.

**Fig. 6:**
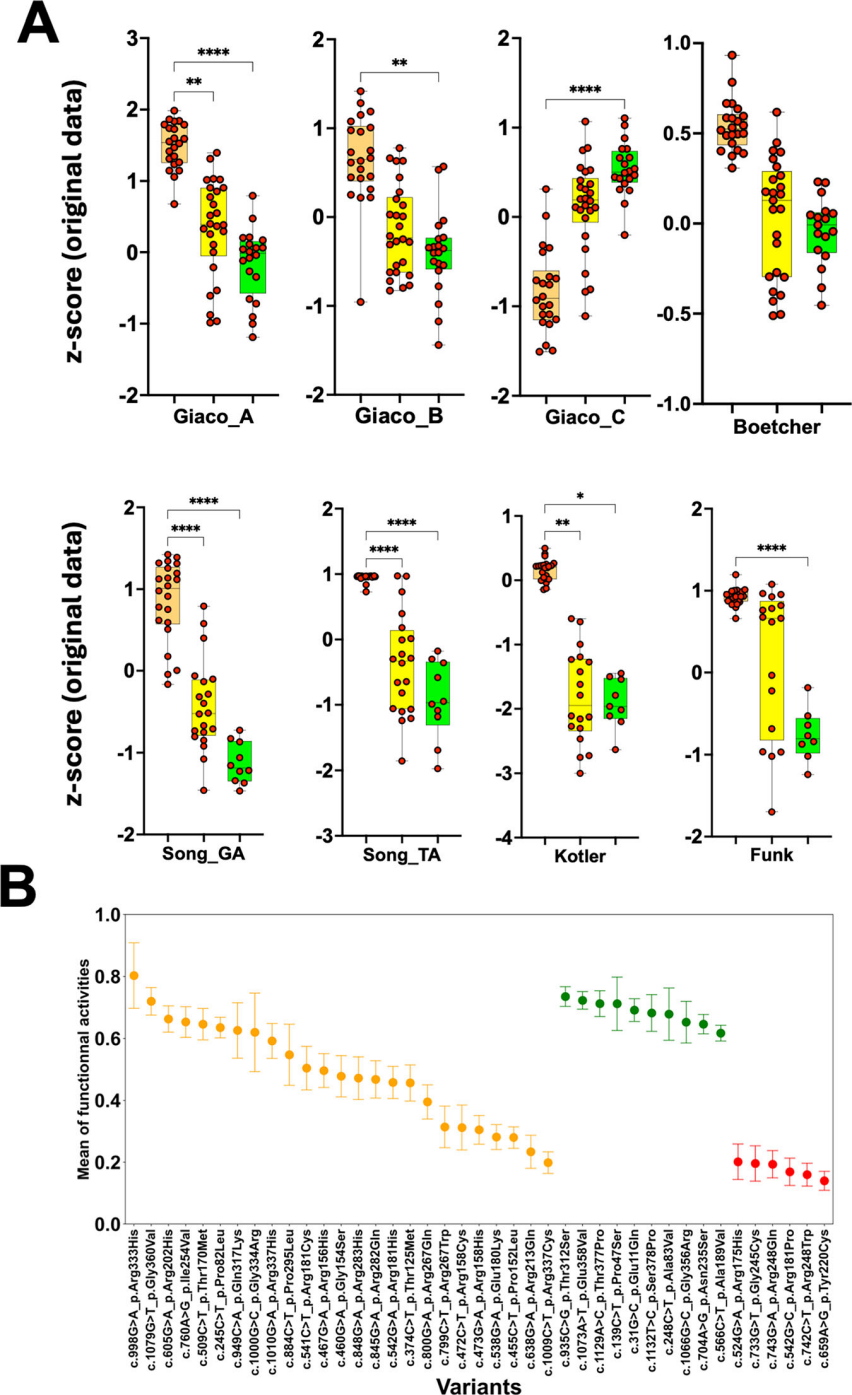
Most h-GVSr p53 variants display partial loss of activity in mammalian cells. **A** Remaining activity of *TP53* variants using MAVE analysis performed in mammalian cells. Green, yellow, and orange box plots correspond to SNPs, hypomorphic and hotspot variants, respectively. In several datasets, data were only available for the DNA-binding domains (residues 100 to 300), precluding information on variants located at the amino- or carboxy-terminus of p53. A detailed analysis of each p53 variant is shown in Supplementary Fig. S8. Original z-scores were taken from each publication as described in “Methods”. **B** Mean and standard error of the mean (SEM) of remaining activity for each p53 variant after functional data normalization with h-GVSr variants in orange, SNPs in green and hotspots in red.

Several multiplexed assays of variant effects (MAVEs) have been performed to analyze any remaining activity of p53 variants (see “Methods”). Although they used different readouts, a correlation analysis showed excellent agreement among them^28^ (Supplementary Fig. S6). The first MAVEs, performed by Kato et al., was based on the transcriptional activity of the variants in a yeast assay on eight different p53 response elements (p53RE)^29^. Although this work did not directly predict any tumor-suppressive effect of p53, it is still considered a good proxy for p53 functionality and has been widely used to predict variant pathogenicity. The distribution of the median value obtained from the analysis of the eight promoters showed that the majority of h-GVSr variants displayed a partial loss of activity, compared to SNPs and hotspot variants (Supplementary Fig. 7A, B). A detailed analysis of all these variants, summarized in Fig. 5, confirmed their mild and heterogeneous loss of activity. Five MAVEs studies on p53 were conducted in mammalian cells, resulting in eight distinct functional datasets. Four of these studies were based on expression data in cDNA libraries of p53 variants. In contrast, the fifth involved the analysis of p53 variants generated by CRISPR mutagenesis of the endogenous *TP53* gene. This allowed for the elimination of overexpression artifacts and the assessment of variants in their native genomic context (see “Methods”). In all the datasets, h-GVSr variants displayed a partial loss of activity compared to SNPs and hotspot variants (Fig. 6A). The trend of loss of activity for hypomorphic variants was similar regardless of the mammalian cell data that were used (Fig. 6A). The normalization of the functional data from all of the studies, yeast and mammalian, for each p53 variant showed a general correlation across the various datasets and confirmed the intermediate phenotype of most h-GVSr variants (Fig. 6B). However, a subset of h-GVSr variants—particularly those with the lowest loss of activity—display functional scores that fall within the range observed for validated benign SNPs. For these variants, the boundary between a hypomorphic allele and a rare, population-specific SNP is inherently ambiguous and cannot be resolved solely on the basis of the current functional data.

The convergence of five independent MAVEs studies in classifying the majority of h-GVSr variants within an intermediate activity range (Figs. 5, 6, and Supplementary Fig. S7) provides robust support for the biological consistency of this intermediate phenotype in most cases. Nevertheless, for a minority of h-GVSr variants showing only marginal reductions in activity, this convergence is less informative: their functional profiles remain statistically indistinguishable from those of benign SNPs, and the assignment to the hypomorphic class rather than the SNP class must be interpreted with caution.

### Attenuated p53 transcriptional signatures in tumors with h-GVSr p53 variants

**Fig. 7:**
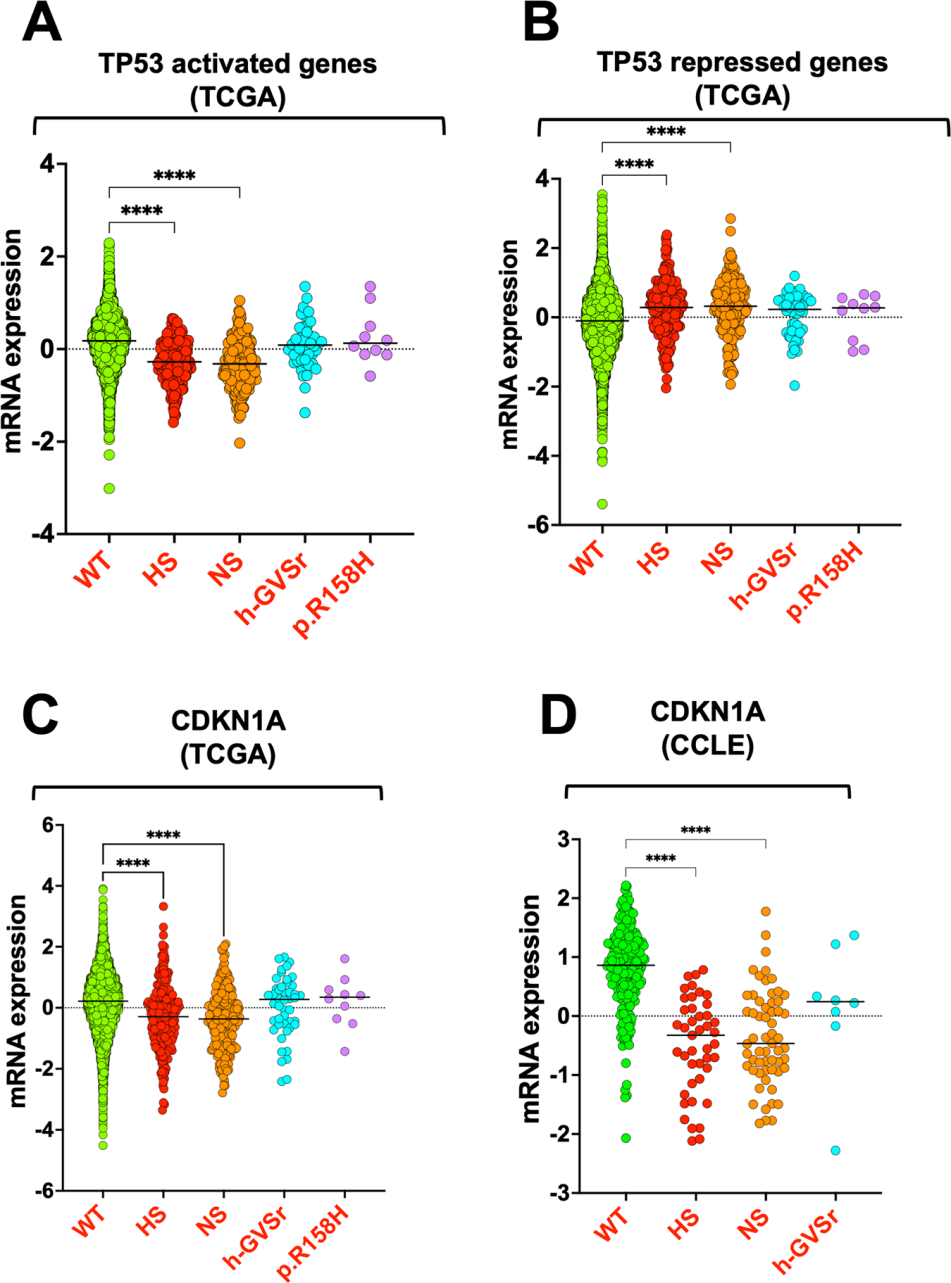
Comparison of RNA expression levels for p53-activated and p53-repressed genes in tumors with various *TP53* mutation types. Analysis was based on data from The Cancer Genome Atlas (TCGA) (**A**–**C**) and The Cell Line Compendium (CCLE) (**D**). Tumors harboring p53 hotspot or nonsense (null) mutations show significantly reduced expression of p53-upregulated (activated) genes (left panel) while also exhibiting derepression and increased expression of p53-repressed genes (right panel). Tumors with hypomorphic *TP53* variants display neither a significant decrease in p53-activated gene expression nor a significant increase in p53-repressed gene expression compared to wild-type or other mutant variants. Data are presented as mRNA expression levels for wild-type (WT), hotspot, nonsense (NS), and h-GVSr *TP53* variants. Data for h-GVSr variant p.R158H found frequently in the TCGA dataset are also presented. RNA expression levels for upregulated (**A**) or repressed (**B**) genes are averaged across 8 and 9 significant p53-regulated genes, respectively. A detailed analysis of each gene is available in Supplementary Fig. S8. Data specific to the CDKN1A gene taken from TCGA or CCLE are shown in (**C**) and (**D**). See “Methods” for further details. *P*-values were calculated using the Kruskal–Wallis test. **, *** and **** indicate *P*  < 0.01, *P* < 0.001, and *P* < 0.0001, respectively. Non-significant (ns) results are not shown.

We next aimed to compare the RNA expression levels of significantly up and downregulated genes in wild-type versus *TP53-*mutated tumors, as reported by the Cancer Genome Atlas (TCGA). Many genes upregulated by p53 are direct target genes, whereas most of those downregulated by it are indirectly controlled via the DREAM repressor complex. To identify genes upregulated, downregulated or unaffected by p53 activation, we used a recently developed strategy that combines transcriptome data associated with the differentiation of murine hematopoietic stem cells (a process correlated with p53 activation) and ChIP-seq data for the binding of p53 or DREAM subunits in murine and human cells^30^. This approach defined three sets of genes: those upregulated directly by p53, those repressed by the DREAM repressor complex, and those unaffected by p53 (Supplementary Fig. S8A). We then compared the RNA expression levels of significantly up and downregulated genes in wild-type versus *TP53-*mutated tumors, as reported by the Cancer Genome Atlas (TCGA) (Fig. 7A, B and Supplementary Fig. 8B–D). Although p53-hotspot or p53-null variants are linked to a significant reduction in the expression of p53-upregulated genes such as CDKN1A, this was not seen with h-GVSr variants (Fig. 7A, C and Supplementary Fig. S8A). Similarly, tumors expressing h-GVSr variants did not display a significant overexpression of p53-repressed genes compared to other variants (Fig. 7B and Supplementary Fig. S8C). No differential regulation was observed for p53-non-regulated genes (Supplementary Fig. S8D). A similar outcome was observed when using transcriptomic data from tumor cell lines (Fig. 7D and Supplementary Fig. S8E). In summary, the functional loss associated with h-GVSr p53 variants in human tumors is consistently intermediate between wild-type and hotspot/nonsense mutations.

### Computational predictions as supporting evidence for TP53 variant classification

Multiple computational tools for predicting p53 loss of function have been developed. These tools leverage evolutionary conservation, structural analysis, and machine learning to assess mutation effects. Recent advances have led to tools employing generative artificial intelligence to predict protein structures (e.g., AlphaFold2) in combination with stability analysis tools (FoldX) to assess loss-of-function impacts on multiple genes. Of note though: purpose-built predictors like AlphaMissense often outperform other approaches. The results from five of the most widely used computational tools showed that h-GVSr variants have intermediate scores falling between those of SNPs and hotspot variants (Supplementary Fig. S9A). Using TP53_PROF, a machine learning model that integrates yeast, mammalian, cell-based and computational metrics, we confirmed that both hypomorphic variants and SNPs were predicted to be either functional or partially functional, compared to hotspot variants that were fully inactive (Supplementary Fig. S9B).

### h-GVSr p53 variants segregate into functional classes associated with variable penetrance

**Fig. 8:**
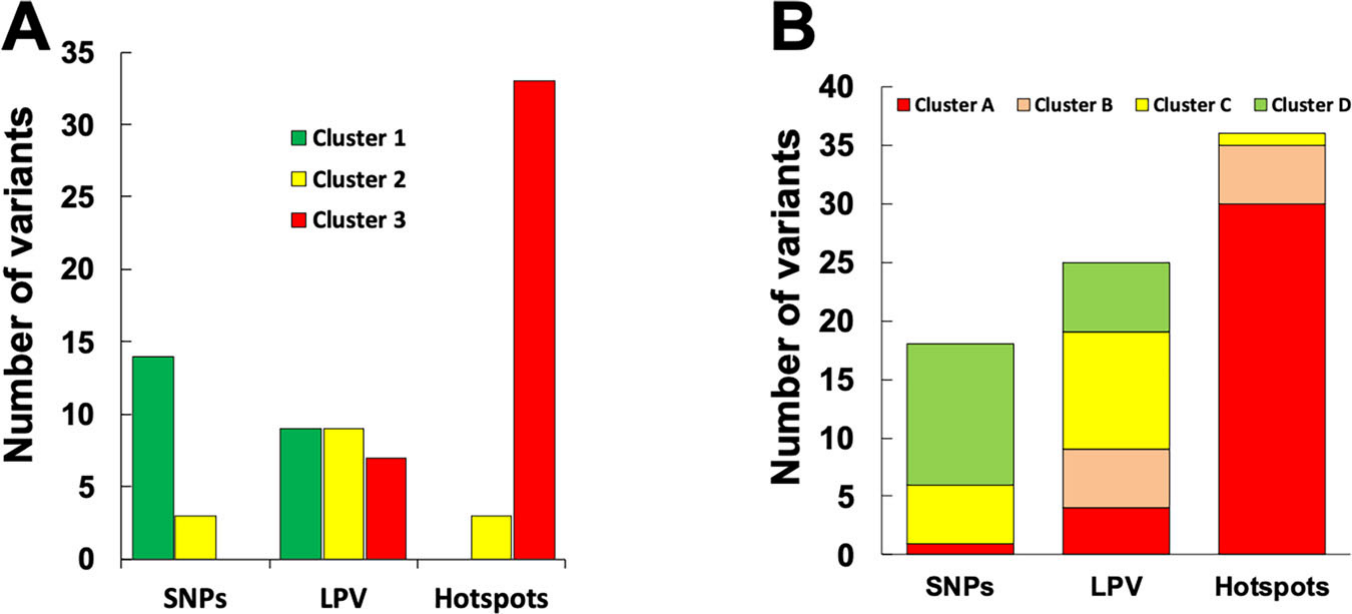
Classification of h-GVSr p53 variants. **A** *TP53* data from the yeast assay were used to cluster p53 SNVs as previously described (Supplementary Fig. S10)^12^. Hotspot variants, such as p.R175H or p.Y220C, are found in cluster 3, which contains the most defective p53 variants. SNPs (p.N235S or p.V31I) are associated with cluster 1, which includes non-defective variants. Most variants in cluster 2 belong to the h-GVSr set. **B** distribution of p53 variants according to the four clusters defined by Montellier et al.^31^.

In our previous clustering analysis, we identified three groups associated with either total, partial, or minimal loss of activity (3, 2, and 1, respectively) (Supplementary Fig. S10). Although most hotspot variants were included in cluster 3, the h-GVSr variants identified in the present study were found preferentially in clusters 2 and 1, the latter comprising SNPs as well (Fig. 8A). Using a different strategy on the same set of functional data, Montellier et al. defined four clusters (A to D, with A indicating the greatest loss of function and D the least) (Fig. 8B)^31^. Furthermore, they showed a strong correlation between disease penetrance in patients with germline variants associated with the different clusters: cluster A was associated with a fully penetrant phenotype, whereas clusters B, C and D showed an attenuated phenotype and a lower lifetime cancer risk. h-GVSr variants were predominantly classified in clusters B to D, which is coherent with our initial finding that these variants are primarily defined by their unbalanced occurrence as germline and somatic as identified by the GVSr analysis. In a recent study, Fortuno et al. identified 11 reduced-penetrance p53 variants associated with partial loss of function. Eight of those variants are among the 26 variants identified by the GVSr analysis (Table S1 and Supplemental Table S2). One variant (c.766 A > G_p.T256A), with a GVSr value of 28, showed a nominal association (Fisher *p* = 0.02) that became insignificant after correction for multiple testing (FDR). The frequency of a second variant in UMD_TP53, c.1101-1 G > A, was below the cut-off used for the analysis (occurrence greater than 20 in the database). However, a second GVSr analysis using a lower cut-off (10) revealed that this variant had a highGVSr (38), validated by the statistical analysis (Supplemental Table S2). This analysis also identified 17 other h-GVSr variants, suggesting that this class of p53 variants is more important than previously thought (Supplemental Table S2).

## Discussion

Using UMD_TP53, currently the largest collection of p53 mutations, we discovered a new group of p53 variants present as both somatic and germline mutations but with a notable bias toward germline origin. Compared to classic hotspot variants, the variants in this new group were found at medium frequencies in human tumors. Further analysis using multiple independent functional or predictive criteria showed that most of these variants were, as compared to fully dysfunctional hotspot variants, hypomorphic with partial loss of function. While the majority of h-GVSr variants are clearly distinct from genuine polymorphisms on the basis of their functional and epidemiological profiles, a minor subset displaying only marginal functional impairment occupies a grey zone in which the distinction from a very rare benign SNP remains difficult to establish with certainty. Additional analysis indicated that when found as somatic in human tumors, these variants were often associated with a second p53 variant, suggesting that they have lowpenetrance, again as compared to entirely dysfunctional hotspot variants. Analysis of transcriptomic data from TCGA revealed that these variants had a weaker impact on mutant *TP53* RNA expression signatures, an observation confirmed by the analysis of RNA data from The Cancer Cell Line Encyclopedia (CCLE) project.

*TP53* variants with neomorphic activities have been intensely investigated, generating contradictory results not yet ready for the clinic. Germline hypomorphic variants associated with low-penetrance cancer risk have received little attention and are surely underestimated. As early as 1999, Varley et al., through the analysis of germline variants in childhood adrenocortical tumors, had suggested that some variants, such as p.R158H or p.P152L—both identified in our work and detected in different unrelated patients—could be low-penetrance^32^. Further study has shown that p.R152L is not associated with an increased risk of breast cancer, but it is associated with an altered tumor penetrance showing a lower cumulative risk of cancer for all non-adrenal tumors when compared to hotspot variants at codon 245 or 248^33^. Variant p.R181H, also identified in the present work, has recently garnered great attention for potential clinical applications. It has been found at high frequency as a germline variant in multiple large-scale studies focusing either on individuals and families with hereditary cancer syndromes, particularly LFS, or hereditary breast cancer^34,35^. An analysis of more than 400,000 individuals identified p.R181H as the most common pathogenic variant^36^. Although less frequent, variant p.R181C has been described as a founder variant in Middle Eastern families associated with an increased risk of breast cancer^19–22^. Another hypomorphic variant identified in the present study, p.R337H, is common in southern Brazil and associated with different levels of cancer risk in both children and adults. Unlike those with high-penetrance *TP53* pathogenic variants, many patients with p.R181H, p.E181C and p.R337H do not develop cancer^17,35^. Mouse models for p.R181C and p.R337H display late tumor onset and incomplete penetrance^15,37^. Among the newly identified hypomorphic variants, pR156H was detected in multiple families not meeting LFS criteria^38^. Two independent reports described apparently unrelated patients from classic LFS families who harbored two germline p53 mutations, p.R156H and p.R267Q, both included in the h-GVSr variant set^39,40^. In one case, the two mutations were found to be on the same allele; in the second case, the configuration was not defined. Functional analysis showed that, individually, each variant was associated with a very weak loss of activity. In contrast, the double variant was entirely dysfunctional, which could explain its high penetrance in these families^39^. To our knowledge, this is the first example of a composite p53 variant alteration with two hypomorphic variants leading to the expression of a fully inactive p53 and a high-penetrant phenotype.

**Fig. 9:**
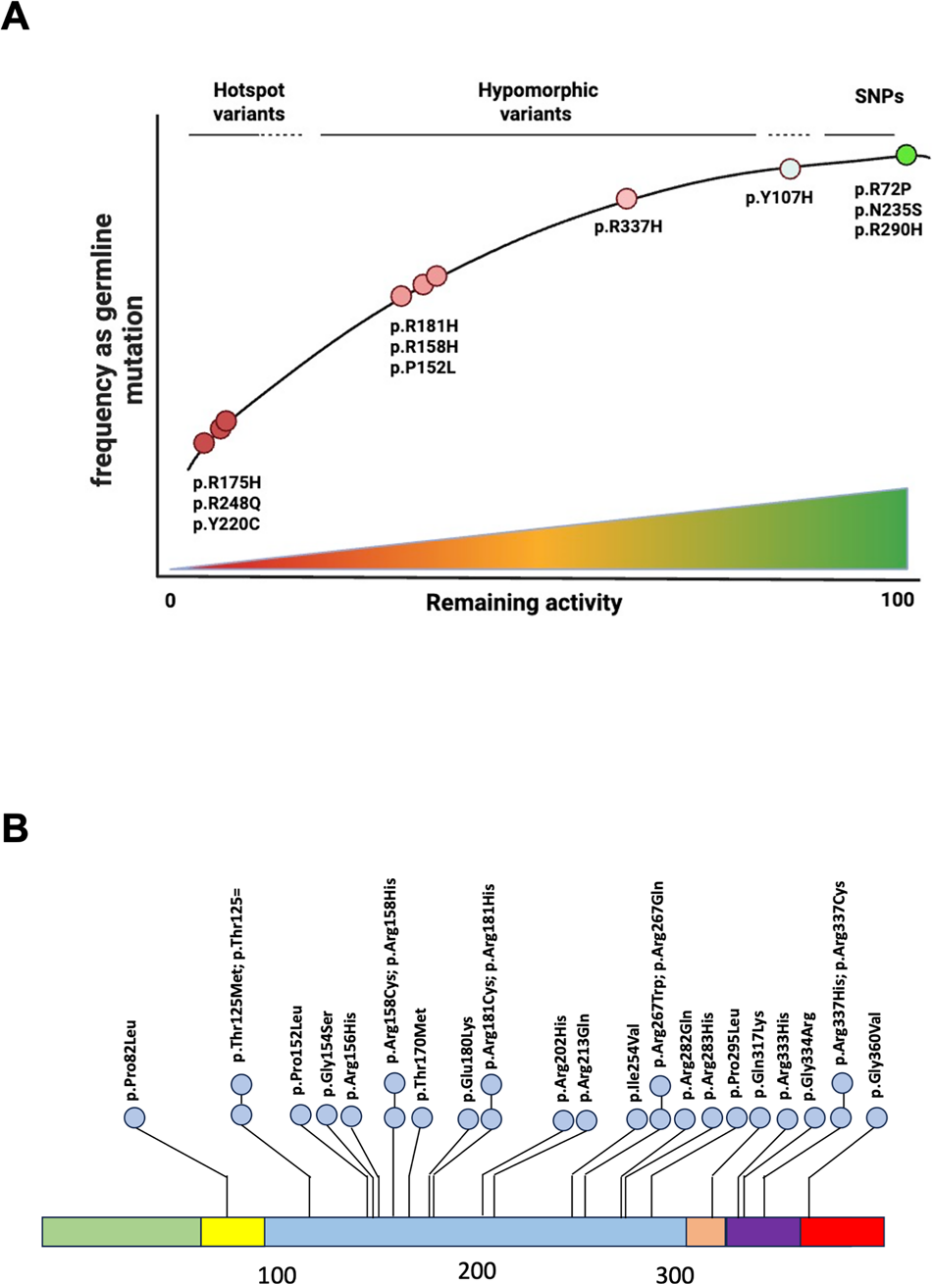
Relationship between the frequency of specific *TP53* variants as germline mutations and their remaining functional activity. **A** The x-axis represents the percentage of remaining *TP53* activity, ranging from 0 (no activity) to 100 (wild-type activity), and the y-axis the frequency of each variant as a germline mutation. Variants are categorized as hotspots, hypomorphic, or SNPs based on their activity and frequency, with hotspot variants showing the lowest activity and highest frequency. Each data point corresponds to a specific *TP53* variant, colored from red (low activity) to green (high activity), to reflect its functional impact. The trend line demonstrates that variants with higher remaining activity are less frequently observed as germline mutations. Variants with lower remaining *TP53* activity, such as hotspot mutations, are associated with higher penetrance as germline variants and typically lead to an earlier onset of cancer. In contrast, variants with higher residual activity often have lower penetrance and are linked to later or less frequent cancer development. Whether or not tumors with low-penetrant variants are associated with better prognosis remains an open question and may differ among the various types of cancer. **B** Landscape of hypomorphic variants along the p53 protein. Green: transactivation domain; yellow: proline-rich region; blue: DNA-binding domain; orange: nuclear localization signal; purple: tetramerization domain; red: negative regulation domain. SNPs are not included in the figure.

The clinical and pathological significance of hypomorphic p53 variants lies in their contribution to a spectrum of cancer predisposition that challenges traditional binary classifications of pathogenicity. These variants exhibit a “functional gradient” that correlates with varying degrees of cancer risk, creating a continuum that extends beyond classic LFS to include lower-risk variants found in the general population (Fig. 9A). This has prompted a shift in terminology and a better understanding of the disease^3,41^. Although LFS was originally described based on a distinctive set of early-onset cancers (such as breast cancer, sarcomas, brain tumors, adrenocortical carcinoma, and leukemia), it is now evident that carriers of germline *TP53* variants face an increased risk of a much wider variety of malignancies, often appearing at unusually young ages depending on the type of variants. To represent this expanded cancer spectrum and the diversity of clinical presentations, a proposal has been made to adopt the term “heritable *TP53*-related cancer syndrome” (hTP53rc syndrome) in addition or as an alternative to Li-Fraumeni syndrome^3^. This terminology would allow inclusion for individuals or families with pathogenic *TP53* variants who do not meet the classic LFS criteria, but still present a significant predisposition to cancer. One limitation of our study is the high stringency of the criteria used to identify hypomorphic variants, which may bias selection toward those found at high frequencies as germline variants. For example, variant p.R175P, which is known to be partially deficient, was not selected for our study because it has never been described as a germline variant. It is therefore likely that other hypomorphic variants, preferentially found as somatic events in tumors, could be identified. Furthermore, the interpretation of hypomorphic p53 variants is further complicated by their interaction with genetic and epigenetic modifiers able to alter their pathogenic potential. For example, the identification of XAF1 p.E134* as a modifier of the p.R337H variant demonstrates how secondary genetic factors can influence cancer risk associated with hypomorphic variants^42^. This finding underscores the importance of considering genetic background when evaluating the clinical significance of hypomorphic p53 variants and suggests that these variants may require additional genetic or epigenetic modifications to fully express their pathogenic potential. Although these variants are found at high frequency as germline variants, the fact that they are also found as somatic events cannot be ignored. This raises a question as to whether these tumors have the same clinical phenotypes as tumors expressing a fully inactive p53 variant.

The results of the present study, based on the analysis of a large dataset of p53 patients included in UMD_TP53, show that these low-penetrant p53 variants are hypomorphic and a non-negligible subset of p53 variants with high clinical value, and that they may represent up to 37% of all non-hotspot p53 variants (Fig. 9A, B)^14^. Nevertheless, there is an inherently fuzzy boundary between very rare benign SNPs and hypomorphic variants with minimal loss of activity. For the subset of h-GVSr variants that retain near-normal p53 function across multiple MAVEs datasets, the distinction from a rare population-specific polymorphism is not absolute. Both categories share high GVSr values and both display near-wild-type functional activity. The discriminating criterion is therefore not functional per se, but epidemiological: true benign SNPs are expected to be detectable at appreciable frequencies in large population databases, whereas low-penetrance hypomorphic variants should remain very rare or absent. While our allele frequency analysis in gnomAD, the Regeneron Genetics Center, the All of Us cohort, and ALFA confirmed that the h-GVSr variants are either very rare or absent in the general population (Supplementary Fig. S2), this distinction will inevitably remain uncertain for variants found only once or twice in these databases. Future studies combining deep population sequencing with co-segregation analyses in cancer-prone families will be essential to definitively resolve the classification of these borderline variants. Hundreds of missense variants found with either germline or somatic origins are currently uncharacterized or misclassified in clinical genetics databases. It is therefore vital to recognize the spectrum of p53 activity loss for accurate risk assessments and the development of personalized cancer treatments, as the level of p53 dysfunction across variants directly affects cancer risk, disease progression, and treatment outcomes^43^. Ultimately, a better characterization of these variants could enable more accurate cancer risk stratification, appropriate surveillance strategies, and improved genetic counseling for individuals carrying p53 hypomorphic alleles.

## Methods

### The UMD_TP53 database

The 2025 release of UMD_TP53 includes 220,177 *TP53* variants gathered from 6127 publications reporting either somatic or germline variants. To avoid redundancy with GENIE, all publications associated with the Memorial Sloan Kettering Cancer Center (MSK) using the MSK-IMPACT targeted sequencing platform were excluded from this study. *TP53* variants derived from cell lines or normal tissues (clonal hematopoiesis) were also omitted. Only missense variants resulting from single-nucleotide variation (SNVs) were retained for all analyses, resulting in a dataset of 116,175 and 7864 somatic and germline variants, respectively. The most common missense *TP53* SNPs, p.P72R (rs1042522) and p.P47S (rs1800371), were removed from all publications and are not included in UMD_TP53. Other, less common SNPs are still included but specifically tagged^23^. The *TP53* variant references (NM_000546.6/NP_000537.3) used in the manuscript correspond to the full-length protein defined by the MANE (Matched Annotation from NCBI and EBI) project^44^.

The AACR Project GENIE data registry (GENIE 17.0-public, released in January 2025) used for validation of p53 complexity was directly downloaded from the GENIE page on Synapse (https://www.synapse.org/Synapse:syn21683345). This version contains 102,706 somatic *TP53* variants from multiple types of cancer^45^.

### *TP53* functional data

The present study used multiplexed assays of variant effects (MAVEs) data from Kato et al.^29^, Kotler et al.^46^, Giacomelli et al.^47^, Boettcher et al.^7^, Song et al.^48^, and Funk et al.^49^.

The transcriptional activity from the work of Kato et al. in yeast transformants containing a p53 cDNA and a green fluorescent protein reporter plasmid was evaluated using eight different promoters for 2314 *TP53* variants. These yeast data are currently the most widely used criteria to define the pathogenicity of *TP53* variants^50^.

Kotler et al. generated a library of *TP53* variants from the core domain of *TP53* (residues 100 to 300) that were analyzed using a range of assays in mammalian cell lines and mice^46^. Only data obtained from the overexpression of 9833 *TP53* variants in the p53-null H1299 cells were used in the present study, as only a subset of their *TP53* variants was used for other read-outs. Data from Giacomelli et al. included 8259 *TP53* variants observed throughout the entire *TP53* protein^47^. The present analysis employed their three different read-outs: *TP53* activity in (i) wild-type A549 cells treated with nutlin, defined as the Giaco_A read-out; (ii) *TP53*-null cells treated with nutlin, defined as the Giaco_B read-out; and (iii) *TP53*-null cells treated with etoposide, defined as the Giaco_C read-out.

Data from Boettcher et al. included 7893 *TP53* variants observed throughout the entire *TP53* protein analyzed in cells expressing wild type p53 treated with nutlin in a setup similar to the Giaco_A experiment. Song et al. analyzed both the transcriptional activity and the growth arrest property of 800 p53 missense variants overexpressed in H1299. Funk et al. performed a deep mutational scan of the p53 DNA-binding domain using saturation genome editing with CRISPR-mediated homology-directed repair to engineer 9225 *TP53* variants expressed in a *TP53* null cell line.

From all these studies, data on *TP53* variants with two or more mutations in different codons, as well as nonsense and frameshift variants, were discarded to focus our analysis on missense variants with a single substitution per codon. The functional data of *TP53* were normalized to a range of 0 to 1, with 0 indicating the lowest activity of *TP53* variants as previously described^28^.

### *TP53* predictive data

Computational data for *TP53* from AlphaMissense^51^, TP53_PROF^52^, Evolutionary Action Score^53^, ClinPred^54^, or BayesDel^55^ were obtained either directly from the publications or their associated webpages. ClinVar data, including FDA-certified information on p53 variants, were obtained in January 2025.

### Population data

*TP53* SNP data have been previously described in detail^13,23^. For the present study, updated data from different population registries were used: Regeneron Genetics Center (https://rgc-research.regeneron.com/me/home), gnomAD (https://gnomad.broadinstitute.org/), the All of Us Research Program of the National Institutes of Health (NIH) (https://www.researchallofus.org/), the NIH Allele Frequency Aggregator (ALFA) database (https://ncbiinsights.ncbi.nlm.nih.gov/2020/03/26/alfa/) and the Single Nucleotide Polymorphism Database (dbSNP), from the National Center for Biotechnology Information (NCBI) https://www.ncbi.nlm.nih.gov/snp/^56–58^.

### RNA expression analysis

The gene expression data (Gene Expression Omnibus #GSE21299) from murine Hoxa9-ER expressing hematopoietic stem and progenitor cells grown in the presence of tamoxifen, or from differentiated cells five days after tamoxifen withdrawal^59^, were analyzed as previously described^30^. Briefly, for each microarray probe, the inverse of Log2 was calculated from robust multi-average values. The obtained average (from triplicates) for cells with tamoxifen was given a value of 1, and the ratios before and after tamoxifen withdrawal were calculated. For each gene, the probe leading to the highest expression fold change upon tamoxifen withdrawal was considered. Relative expression data were graphed with Microsoft Excel, by using a three-color scale and conditional coloring.

The peak browser from ChIP-Atlas (https://chip-atlas.org/peak_browser)^60^ was used to search for Trp53, E2f4, and Lin9 binding on the Mus musculus (mm10) genome, or for TP53, E2F4, and LIN9 binding on the Human genome build 38 (hg38) genome, and results were visualized on the Integrative Genomics Viewer (IGV_2.12.2)^61^. Peaks from all cell types were analyzed, and average ChIP values for regions with the highest peaks and minimal distances from the transcription start site were calculated (after omitting data from stem cells or cells expressing a mutant p53). For each gene, the E2F4/LIN9 ChIP binding score was determined by averaging the E2F4 and the LIN9 scores. ChIP binding scores were graphed with Microsoft Excel using a two-color scale and conditional coloring. Normalized global RNA expression data files for TCGA or CCLE datasets were downloaded from cBioPortal^62^. For each p53-regulated gene or control, RNA expression associated with p53 hotspot variants, p53 nonsense variants, or the h-GVSrs was analyzed.

### Statistical analyses

Statistical analyses, including paired Kruskal–Wallis and Mann–Whitney tests, were performed using GraphPad Prism 10 (GraphPad Software). *P* < 0.05 was reported as statistically significant. All analyses were carried out with Python using pandas (v.2.3.3), NumPy (v.2.3.5), SciPy (v.1.16.3), statsmodels:0.14.5. Plots were generated using matplotlib (v.3.4.2) or seaborn (v.0.11.1). Plots were generated using matplotlib (https://github.com/matplotlib/matplotlib, v.3.4.2) or seaborn (https://github.com/mwaskom/seaborn, v.0.11.1).

## Supplementary information

Sup_table_S1

Sup_table_S2

Supplementary Information

## Data Availability

All data used were either publicly available, as indicated in the references, or are provided within this article and its supplementary information. Data are available on request from the corresponding author, thierry.soussi@igp.uu.se.
