# Supplementary material for: Low-penetrance TP53 variants are mainly hypomorphic: an underestimated issue with high clinical significance
Source: NPJ Genom Med. 2026 Apr 18;11:22. doi: 10.1038/s41525-026-00568-x (PMC13103405; doi:10.1038/s41525-026-00568-x)
Supplement: Supplementary file 3 — Supplementary Information [file 41525_2026_568_MOESM3_ESM.pdf]

**Supplementary document**

# Low-penetrance TP53 variants are mainly hypomorphic: an underestimated issue with high clinical significance.

Lea Rodriguez[1,2], Bernard Leroy[1], Franck Toledo[1], Julianne Susanne Funk[3,4], Thorsten Stiewe[3,4], Panagiotis Baliakas[5,6] François Delhommeau[1,2] and Thierry Soussi[1,2,5,6,*]

1 Sorbonne Université, INSERM, Centre de Recherche Saint-Antoine, CRSA, UMRS_938 Hematopoietic and Leukemic Development, AP-HP, SIRIC CURAMUS, Paris, France

2 Carnot OPALE Institute, F-75010 Paris, France

3. Institute of Molecular Oncology, Universities of Gießen and Marburg Lung Center (UGMLC), Member of the German Center for Lung Research (DZL), Philipps-University, Marburg, Germany

4 Institute of Lung Health, Justus Liebig University, Gießen, Germany

5 Department of Immunology, Genetics and Pathology, Uppsala University, Sweden

6. Clinical Genomics Uppsala, Science for Life Laboratory, Uppsala University, Uppsala, Sweden

* **Corresponding author:** Thierry Soussi

Address: Department of Immunology, Genetics and Pathology, Uppsala University, Uppsala, Sweden

Phone: +46 70 226 12 04

E-mail: thierry.soussi@igp.uu.se

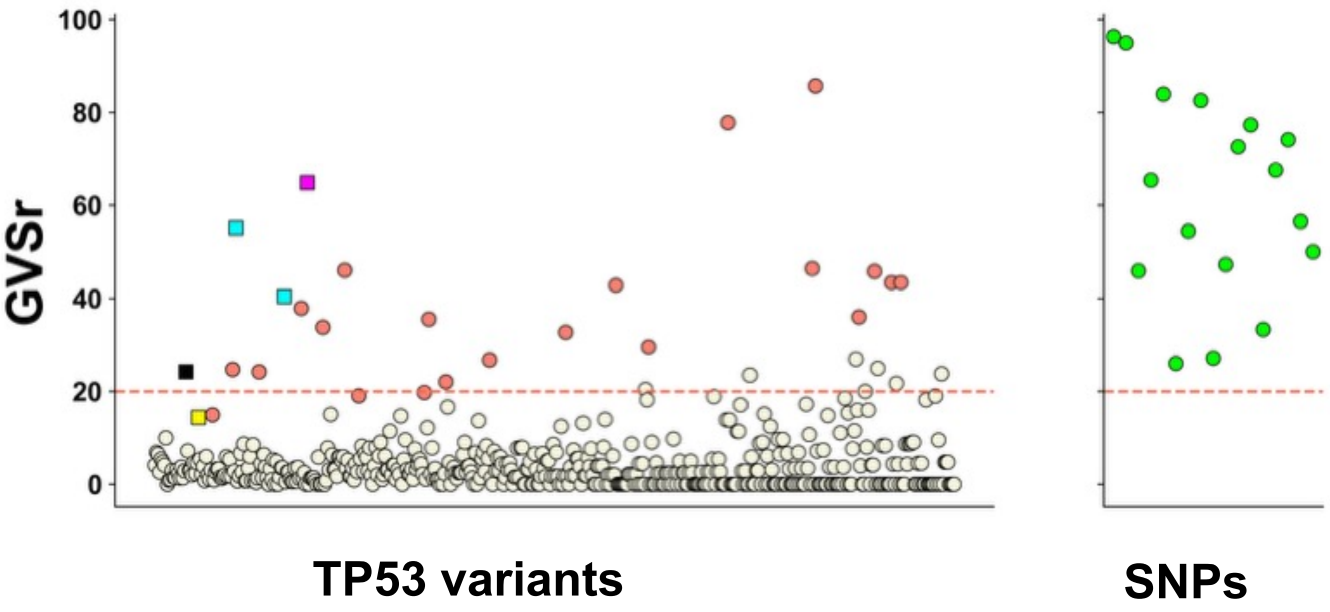


**Supplementary Figure S1: Germline-versus-somatic ratio (GVSr) of *TP53* variants in UMD_TP53**. Left: analysis was performed on missense *TP53* variants with at least 20 occurrences in the database. Variants are arranged from left to right by frequency in the database. Right: The validated missense SNPs identified by Doffe *et al*. are shown in green in the right part of the figure [1]. Hypomorphic variants at codons 181, 152, 158 and 337 are shown in blue, yellow, black, and purple, respectively. Most h-GVSr variants have a GVSr greater than 20 (red line)

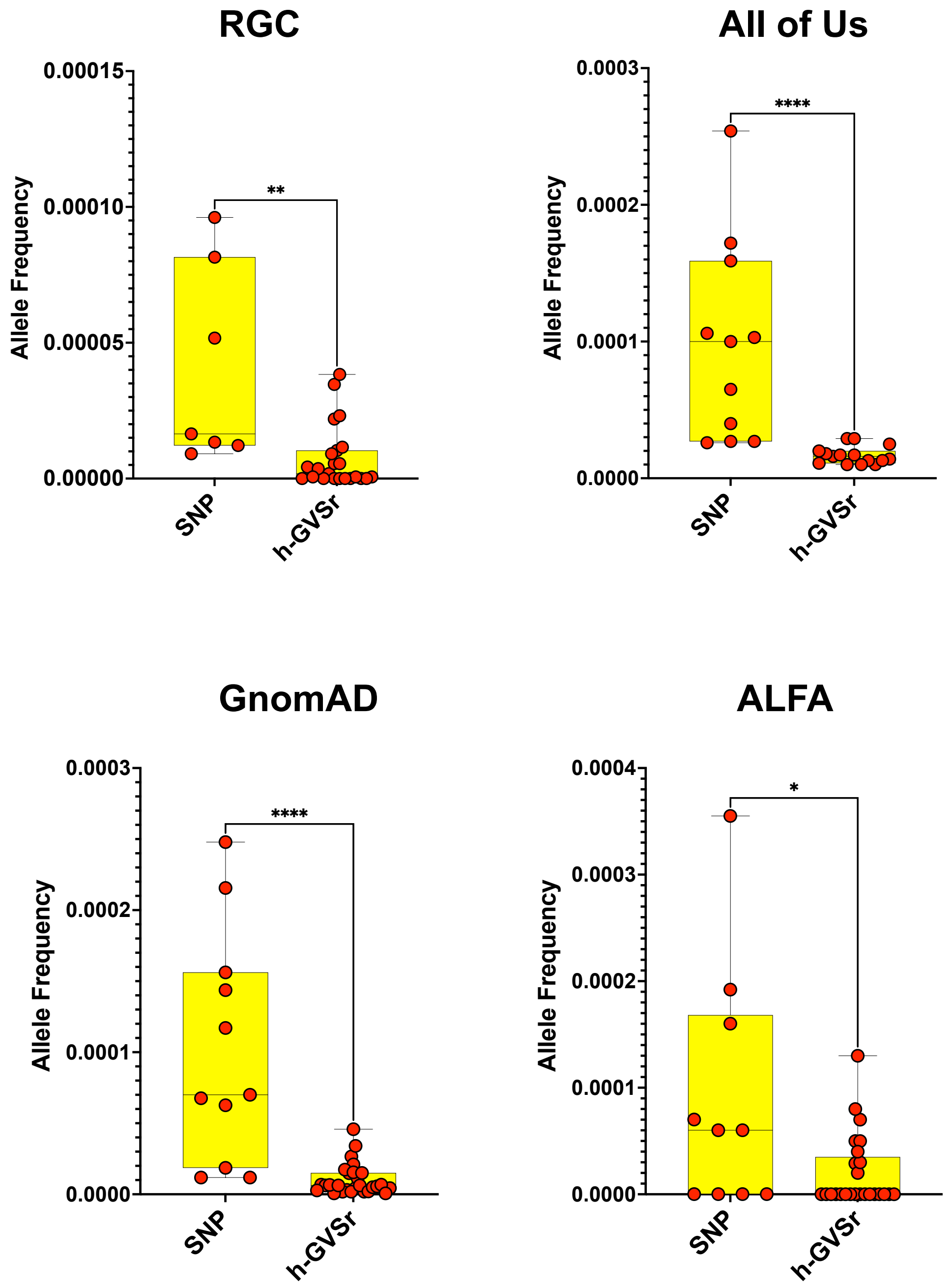


**Supplementary Figure S2: h-GVSr *TP53* variants are very infrequent in human population registries**. *TP53* variant allele frequencies were retrieved from RGC, All of Us, ALFA and GnomAD datasets. P-values were calculated by the Mann-Whitney test. **, *** and ****: P<0.01, p<0.001 and P<0.0001 respectively. (see Materials and Methods for more information about each dataset).

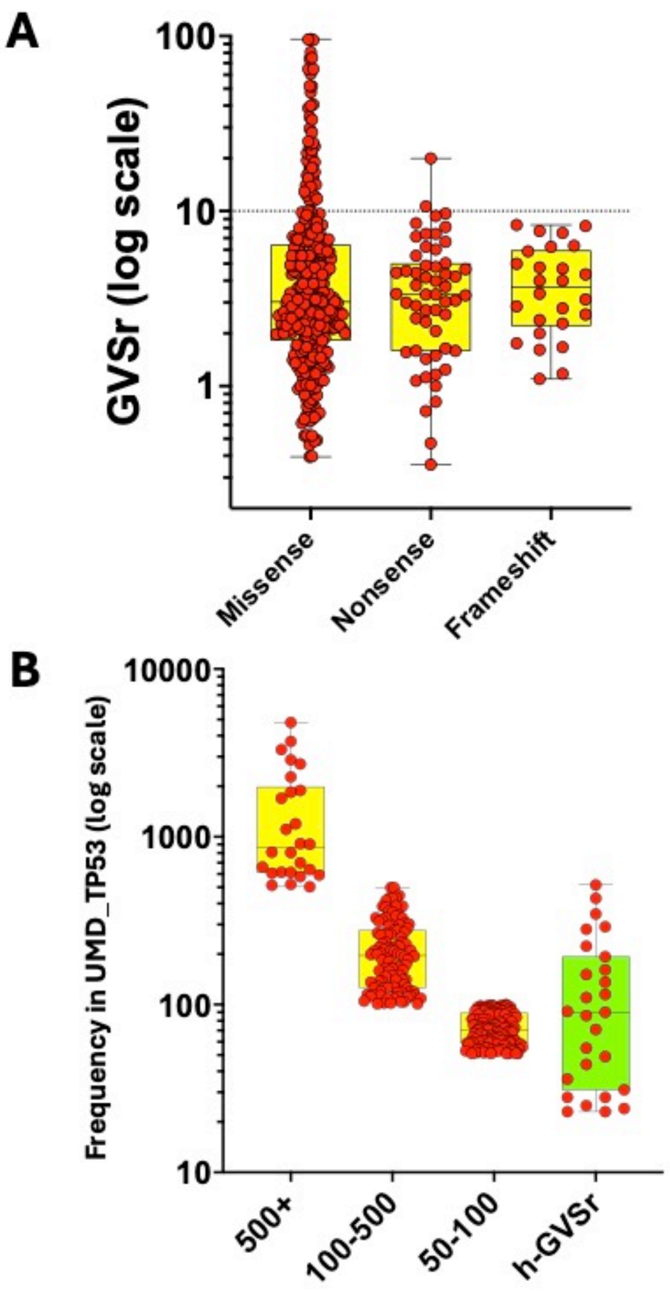


**Supplementary Figure S3: High GVSr is specific for missense variants found at medium frequencies in UMD_TP53**. A: *TP53* variants were sorted into three classes according to their frequency in UMD_TP53 (yellow boxes) and compared to hypomorphic variants (green box). B: Box plots of GVSr from missense, nonsense and frameshift variants at frequencies >50 in UMD_TP53 were analyzed. Only two nonsense variants have a GVSr value greater than 10 (p.Y126* and p.Y103*).

**Supplementary Table S2: Rank of *TP53* variants.**

Table S2A: *TP53* SNVs ranked according to their occurrences as somatic variants in UMD_TP53.

Table S2B: *TP53* SNVs ranked according to their occurrences as germline variants in UMD_TP53. *TP53* variants in set_46. Hypomorphic variants at codons 152, 158,181 and 337 are shown in yellow, black, blue and purple, respectively. SNPs are shown in green.

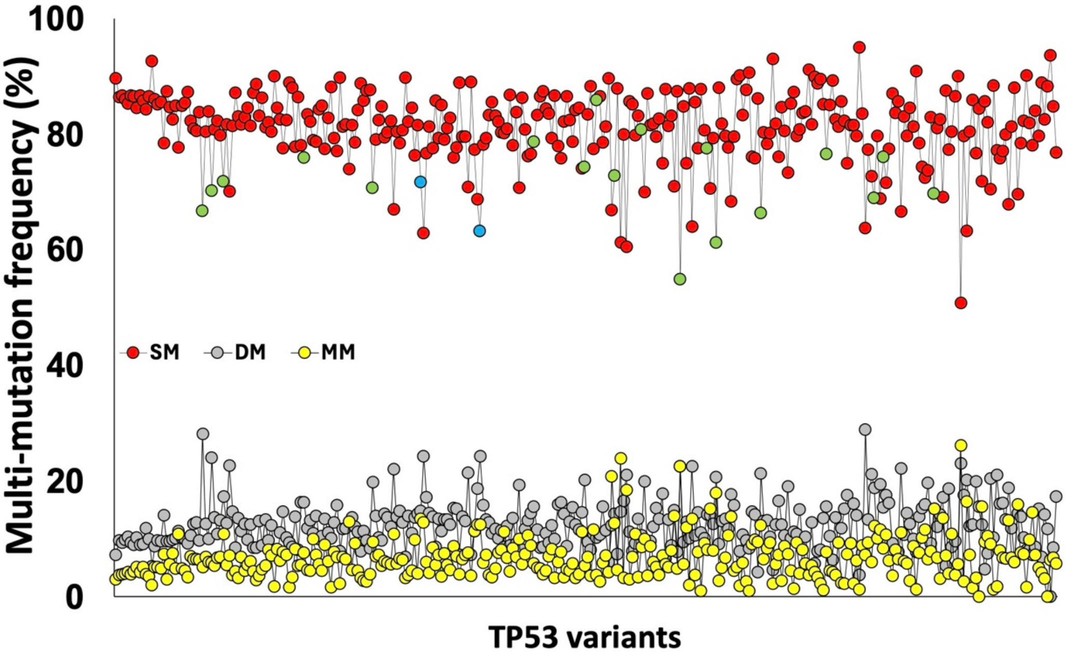


**Supplementary Figure S4: Complexity of *TP53* variants in human tumors.** The frequency of each p53 variant's occurrence as a single mutation in a tumor (SM, red), or associated with a second (DM, grey) or more than one other variant (MM, yellow) is shown. The analysis was performed on the 500 most frequent missense variants in UMD_TP53 (from left to right on the x-axis). Green dots: SNPs and h-GVSr variants; blue dots: variants at codon 181.

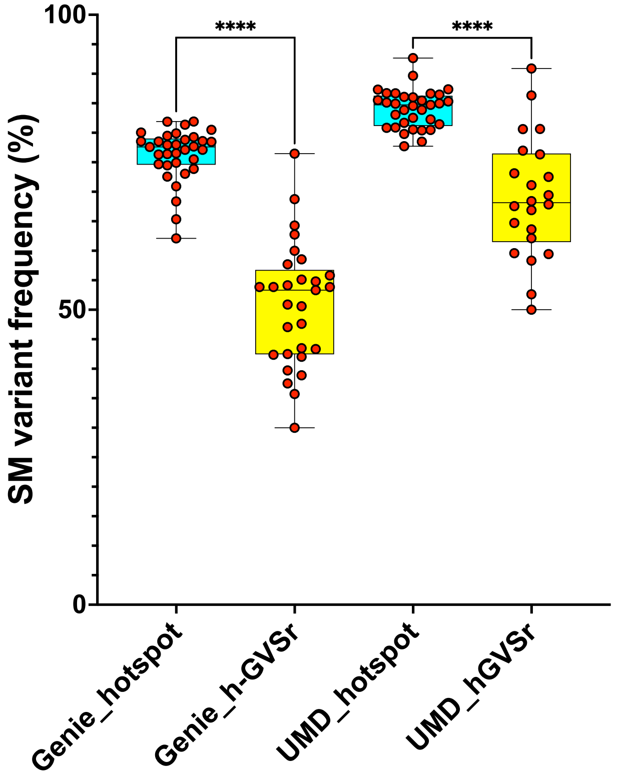


**Supplementary Figure S5: h-GVSr TP53 variants from GENIE have higher p53 complexity compared to hotspot variants**. The frequency of each p53 variant's occurrence as a single mutation (SM) in a tumor is shown. The analysis was performed on the 500 most frequent missense variants in UMD_TP53 or in GENIE. P-values were calculated by the Mann-Whitney test. ****: P<0.0001.

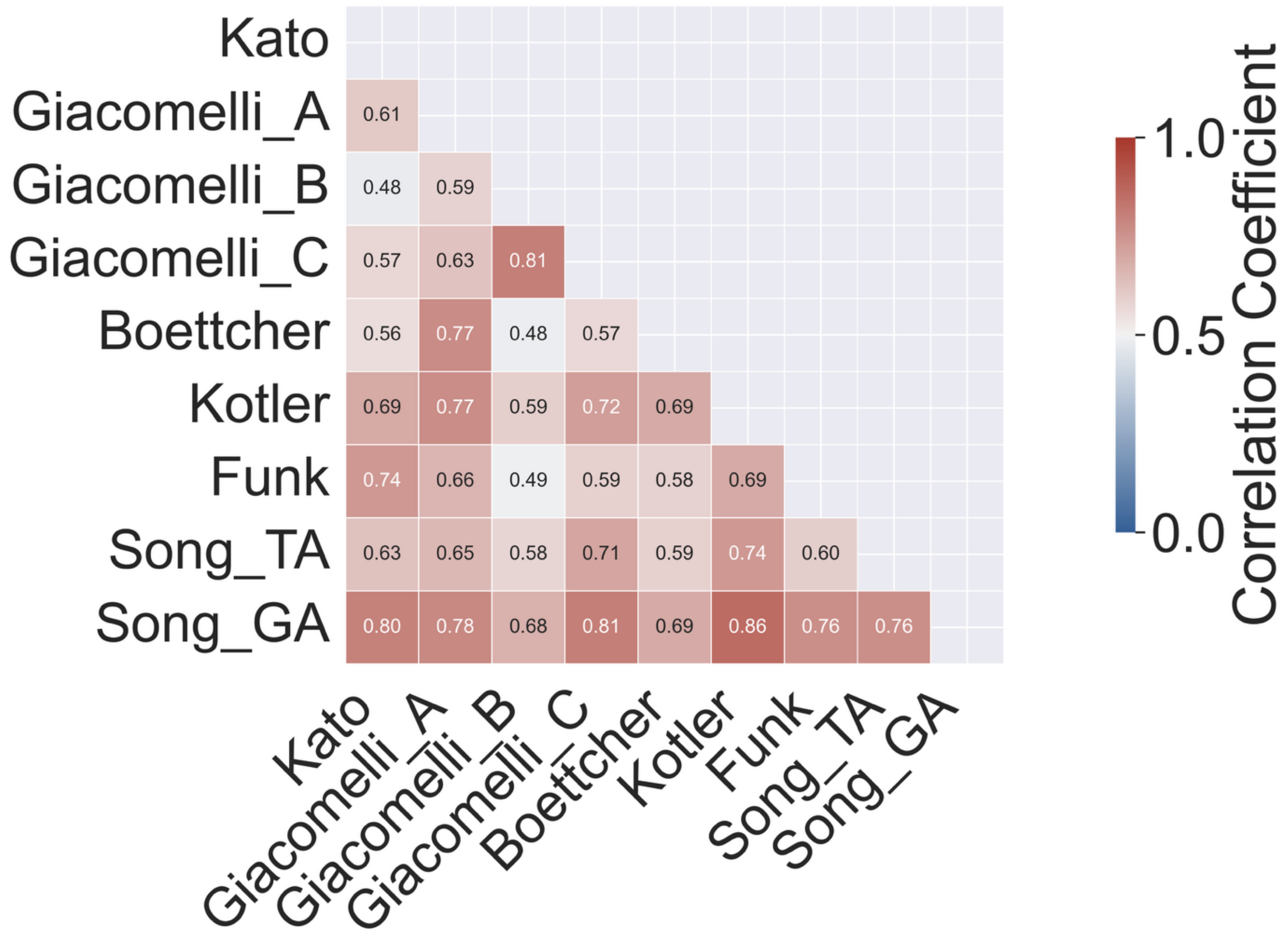


**Supplementary Figure S6:** Correlation matrix for the various MAVEs studies performed on p53 variants. The heatmap displays pairwise Pearson correlation coefficients between p53 variant activity scores from the current study and five previously published high-throughput saturation mutagenesis screens (Song; TA: transactivation, GA: growth arrest). Giacomelli data A, B and C have been defined in Materials and Methods. Values indicate the strength of correlation, with higher values (closer to 1.0) representing greater concordance between functional assays. See Material and Methods for more information on each study.

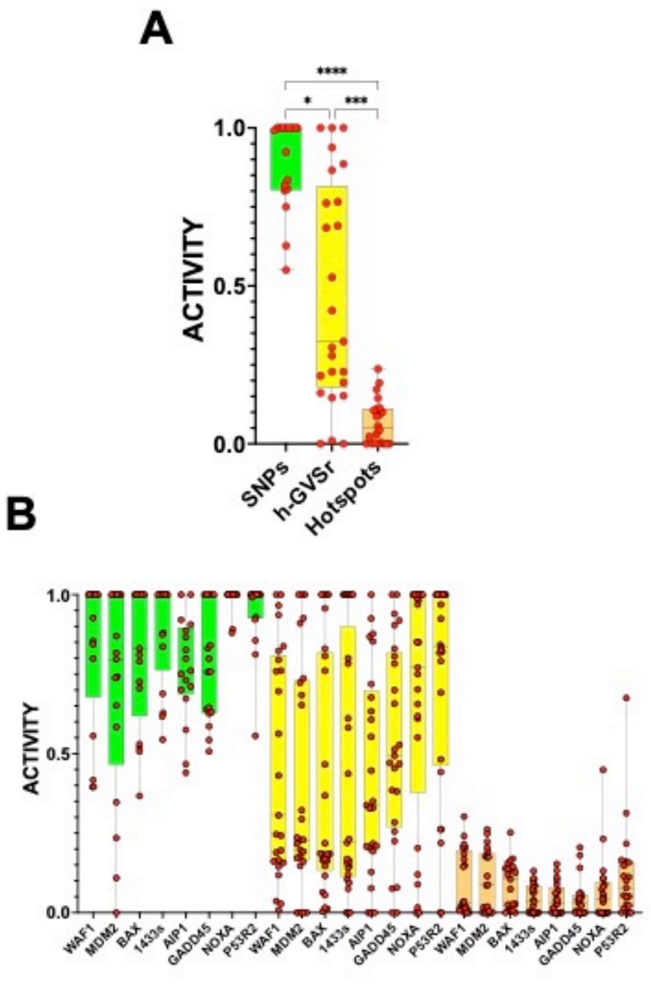


**Supplementary Figure S7: Remaining activity of *TP53* variants using yeast functional data.** A: Global analysis using the functional data (median) on the eight p53 response elements (p53RE). B: Detailed analysis for each of the eight p53RE. Green, yellow, and orange box plots correspond to SNPs, hypomorphic and hotspot variants, respectively. P-values were calculated by the Kruskal-Wallis test. **, *** and ****: p>0.01, P<0.001 and P<0.0001 respectively.

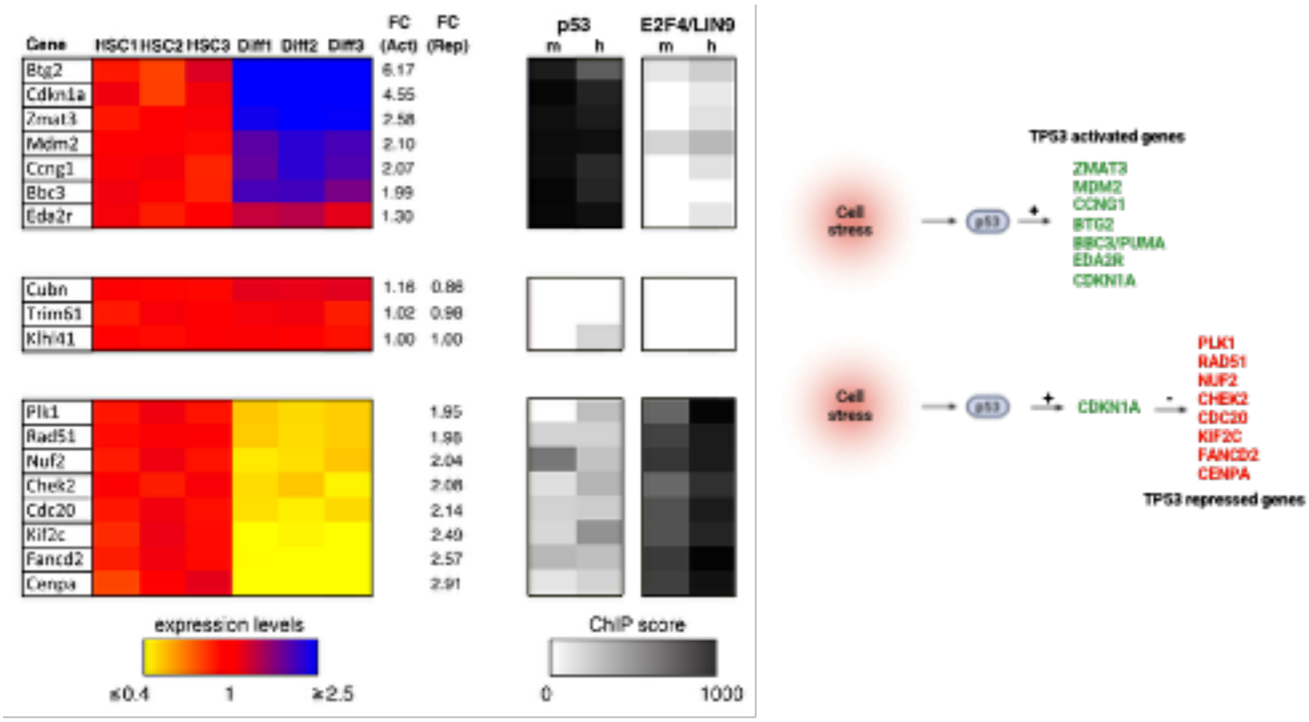


**Supplementary Figure S8A**

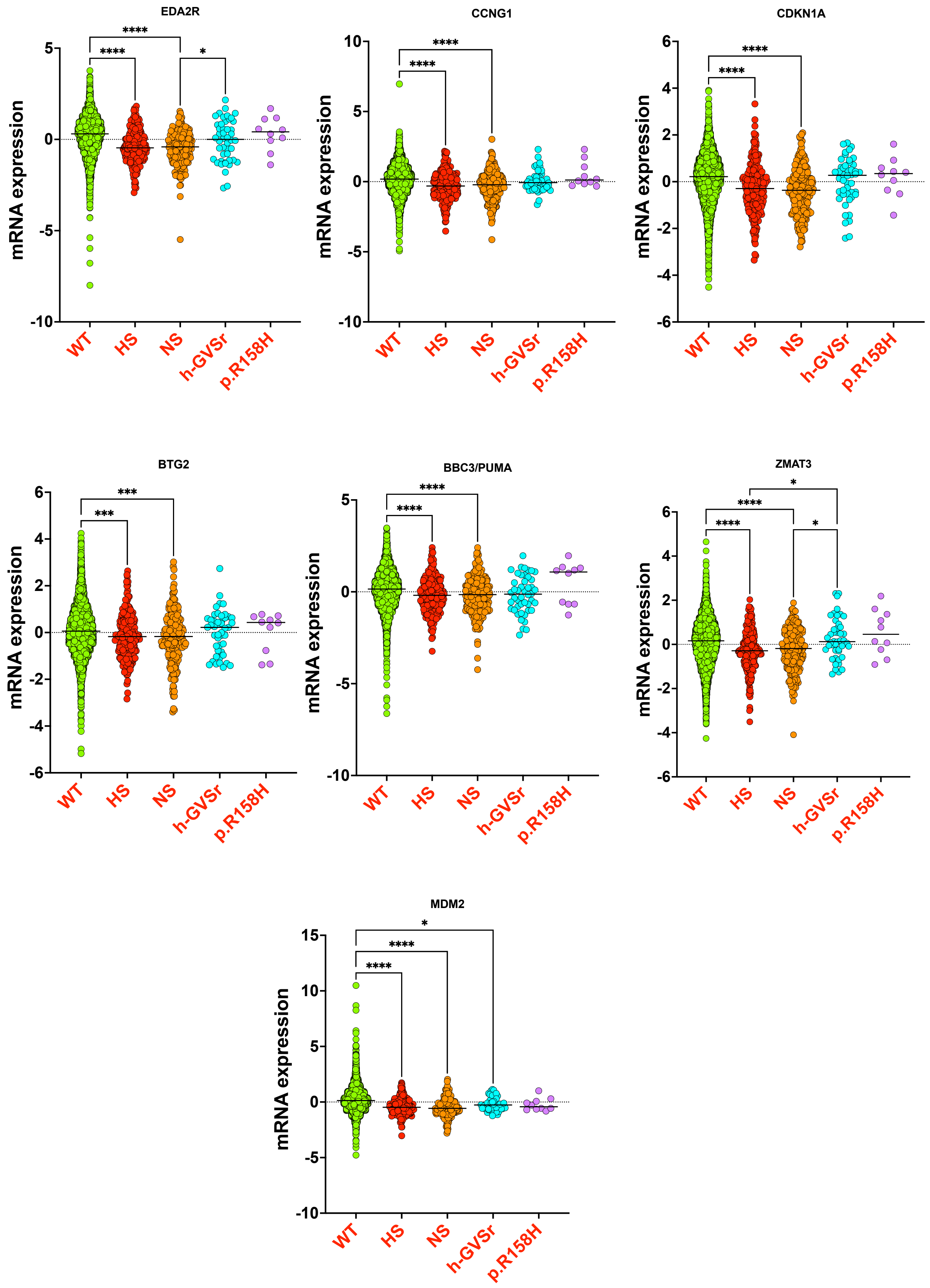


**TCGA activated genes**

**Supplementary figure S8B**

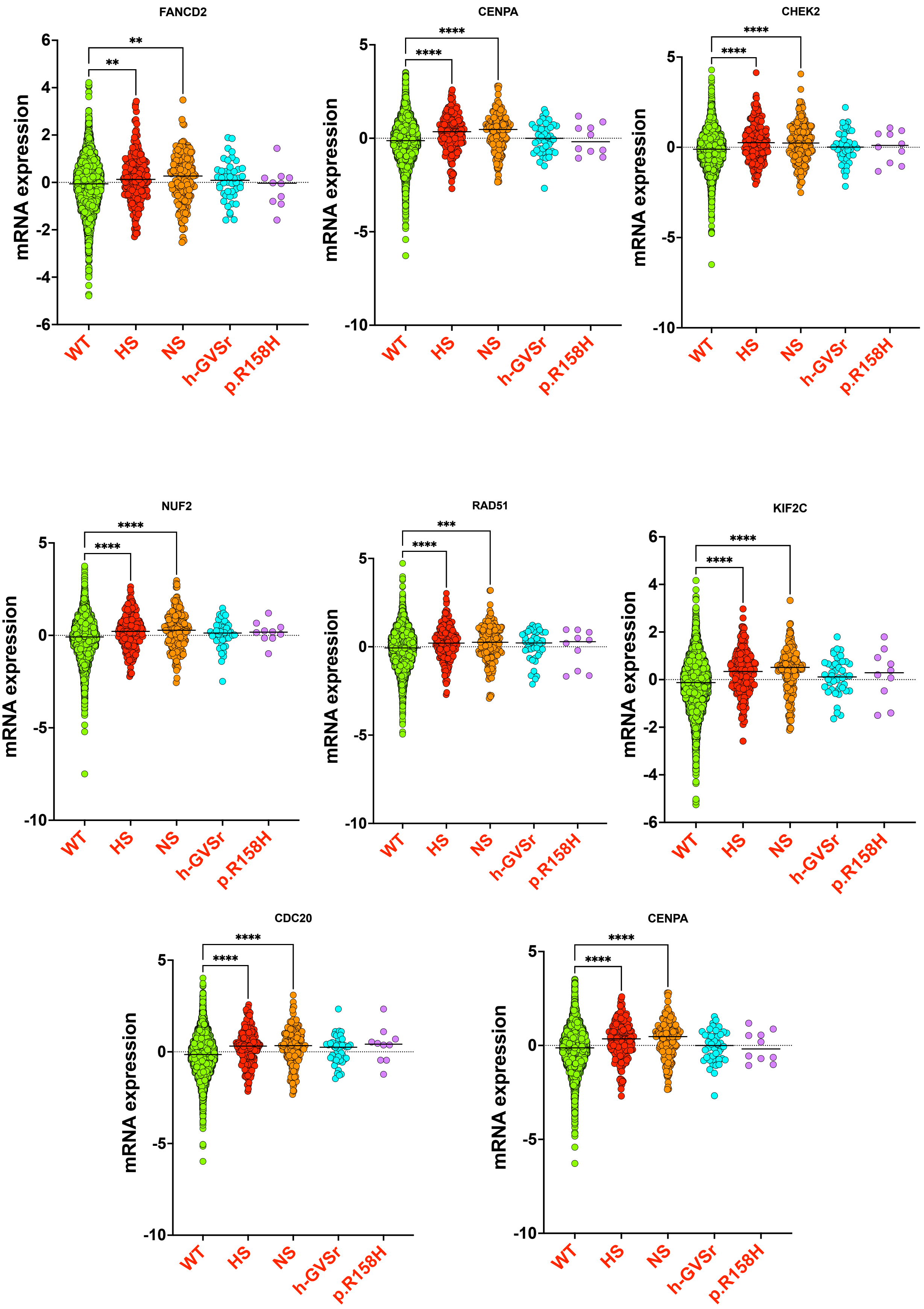


**Supplementary figure S8C**

**TCGA repressed genes**

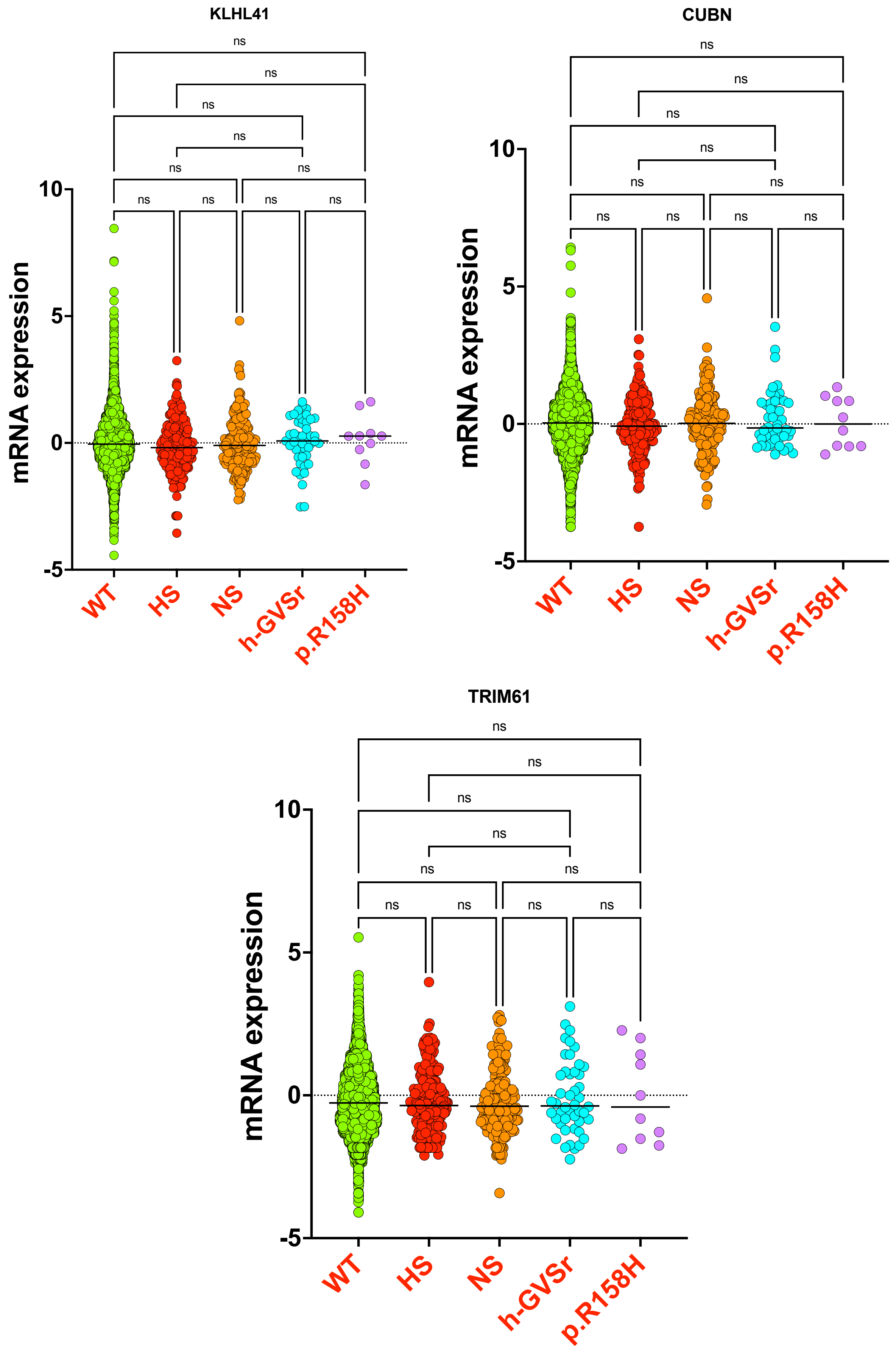


Supplementary figure S8D

TCGA control genes

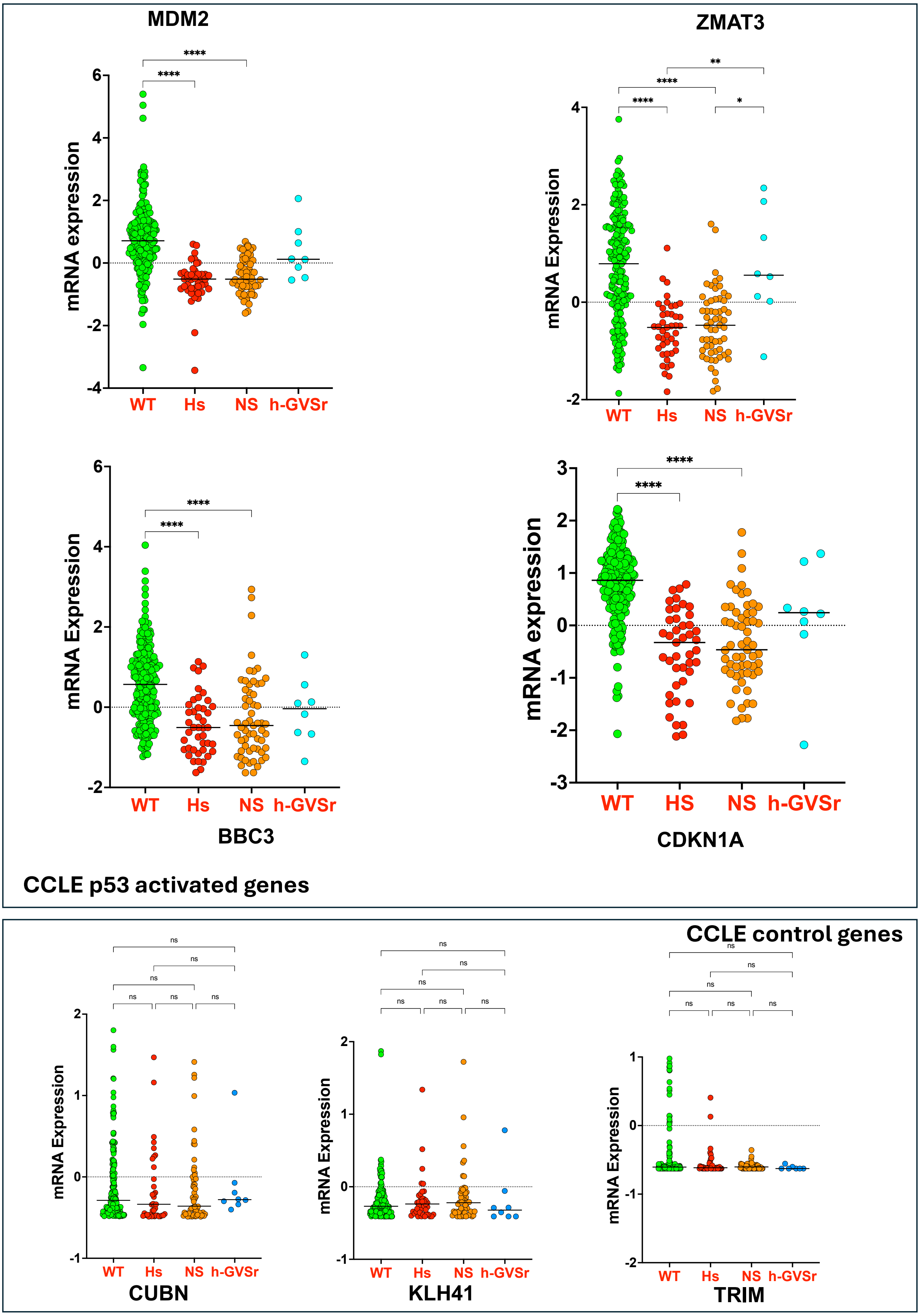


Supplementary figure S8E

**Supplementary Figure S8A to E**

Comparison of RNA expression levels for p53 activated and repressed by the p53-DREAM pathway in tumors with various *TP53* mutation types, based on data from The Cancer Genome Atlas (TCGA) (B and C) and The Cell Line Compendium (CCLE) (D).

A: Left, robust multi-average values for the indicated genes were extracted from transcriptome data (GSE21299) of Hoxa9-ER expressing hematopoietic stem and progenitor cells (HSC) grown in the presence of tamoxifen, or from differentiated cells (Diff) five days after tamoxifen withdrawal (Average values (from triplicates) in cells with tamoxifen were given a value of 1). Genes are listed according to decreasing activation (Act) or increasing repression (Rep) fold changes (FC).Center, the average chromatin immunoprecipitation (ChIP) binding of p53 or the DREAM subunits E2F4 and LIN9 in mouse (m) or human (h) cells are represented. Values were calculated from ChIP-Atlas data (https://chip-atlas.org). Right: illustration of the p53-mediated regulation for activated (top) and repressed (bottom) genes.

B to E:Tumors (B to D) or cell lines (E) harboring p53 hotspot or nonsense (null) mutations show significantly reduced expression of p53-upregulated (activated) genes  while also exhibiting derepression and increased expression of p53-repressed genes . Tumors with hypomorphic *TP53* variants display neither a significant decrease in p53-activated gene expression nor a significant increase in p53-repressed gene expression compared to wild-type or other mutant variants. Data are presented as mRNA expression levels for wild-type (WT), hotspot, nonsense (NS), and h-GVSr *TP53* variants. Data for h-GVSr variants R158H found frequently in the TCGA dataset are also presented.P-values were calculated using the Kruskal–Wallis test. **, *** and **** indicate $p>0.01$, $P<0.001$, and $P<0.0001$ respectively. Non-significant (ns) results are not shown except for panel S8D.

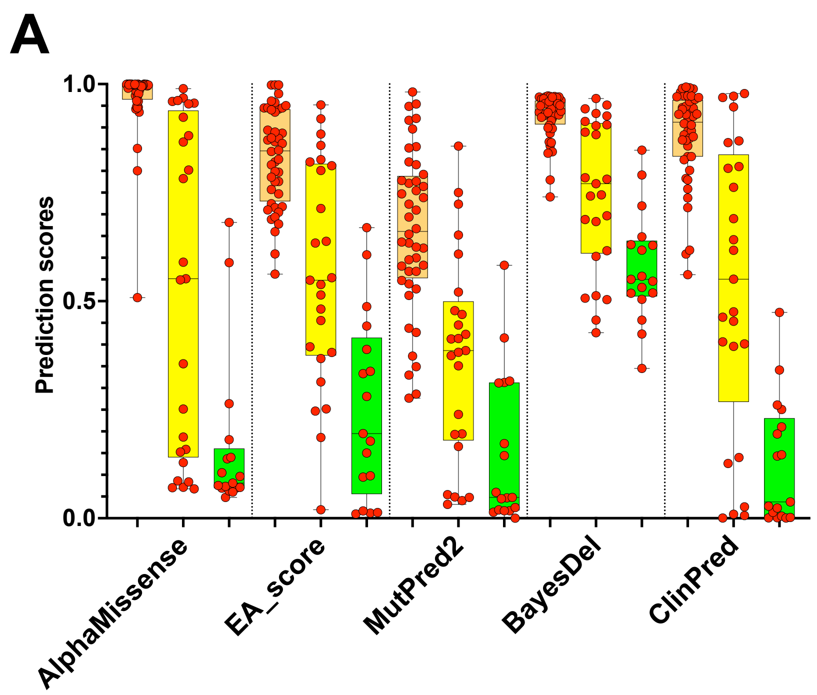


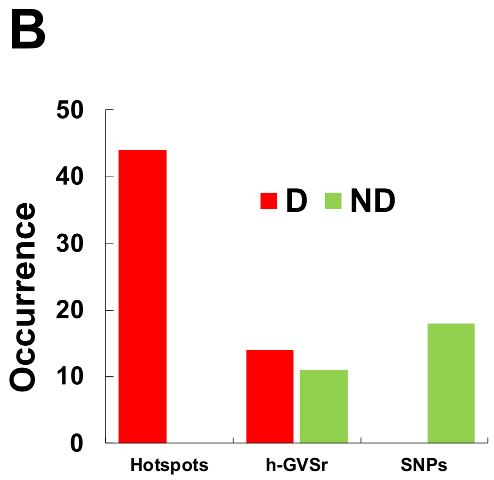


**Supplementary Figure S9: Predictive analysis of p53 variants**. A: Computer score classification of the three datasets. SNPs, hypomorphic and hotspot variants are shown in green, yellow and orange, respectively. Normalized scores range from 0 (predicted to be functional) to 1 (predicted to be nonfunctional). B: p53 classification N (non-deleterious) and D (deleterious) based on *TP53* data from a machine learning analysis of multiple p53 datasets. See Materials and Methods for more information regarding each computer tool.

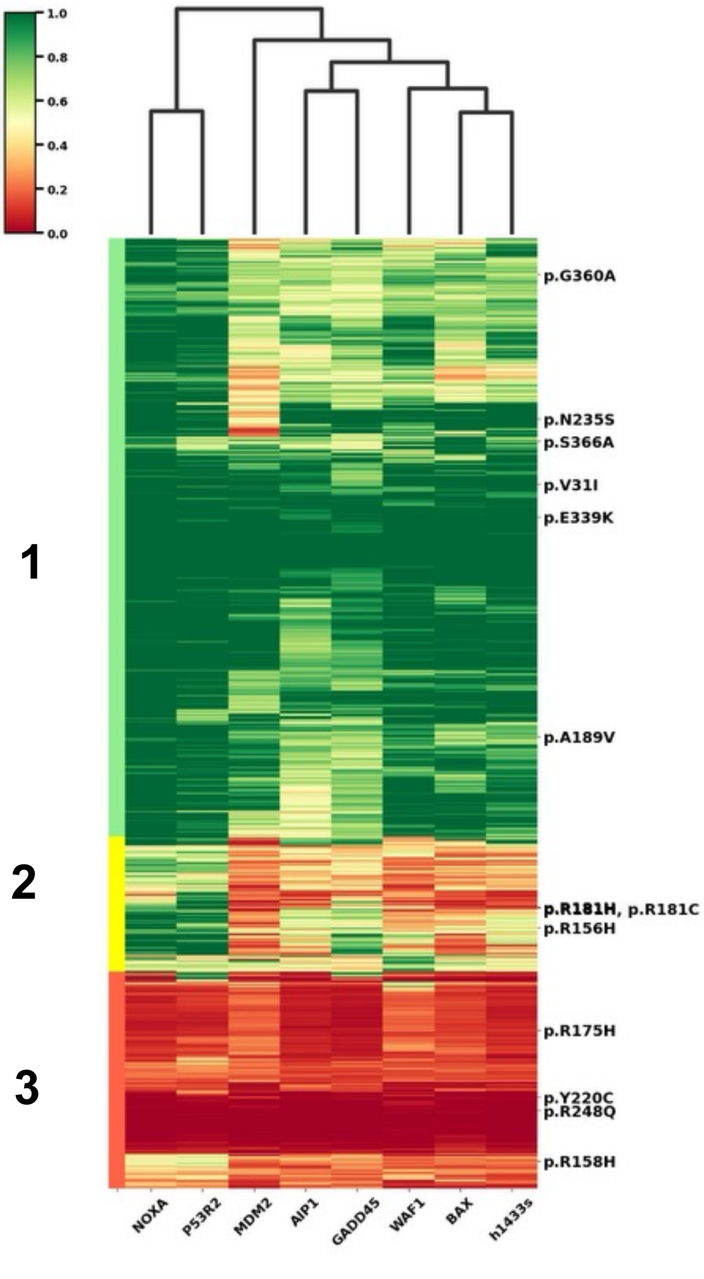


**Supplementary Figure S10: Classification of *TP53* variants**. *TP53* data from the yeast assay were used to cluster p53 SNVs as previously described [2]. Hotspot variants, such as R175H or Y220C, are found in cluster 3, which contains the most defective p53 variants. SNPs (N235S or V31I) are associated with cluster 1, which includes non-defective variants. Partially defective variants are found in cluster 2.

**References**


1. Doffe F, Carbonnier V, Tissier M, Leroy B, Martins I, Mattsson JSM *et al.* Identification and functional characterization of new missense SNPs in the coding region of the TP53 gene. Cell Death Differ.2021; 28:1477-1492

2. Soussi T, Kato S, Levy PP, Ishioka C Reassessment of the TP53 mutation database in human disease by data mining with a library of TP53 missense mutations. Hum Mutat.2005; 25:6-17
